# TGM2 Aggravates Acute Pancreatitis by Impairing Macrophage Efferocytosis Through Inhibition of the STAT6–GAS6 Axis

**DOI:** 10.1002/advs.202520739

**Published:** 2026-02-17

**Authors:** Xuxu Liu, Zhiwei Du, Liyi Wang, Zhihong Xie, Ziang Meng, Yi Zheng, Heming Wang, Yuanhang He, Dongbo Xue

**Affiliations:** ^1^ Key Laboratory of Hepatosplenic Surgery Ministry of Education The First Affiliated Hospital of Harbin Medical University Harbin China

**Keywords:** acute pancreatitis, efferocytosis, GAS6, lipid nanoparticles, TGM2

## Abstract

**Background:**

Acute pancreatitis (AP) is characterized by dysregulated inflammation, with macrophage dysfunction (impaired efferocytosis, pro/anti‐inflammatory phenotype imbalance) exacerbating the disease. Current therapies are mostly supportive, highlighting the critical need for targeted interventions.

**Methods:**

Transglutaminase 2 (TGM2) was identified via public transcriptomic analysis. Its function was validated in caerulein‐induced AP mice and in vitro cell models; mechanisms were explored via Co‐IP, ChIP, and dual‐luciferase assays. A lactoferrin‐modified, ROS‐responsive LF‐LNP system was developed for TGM2 siRNA delivery.

**Results:**

TGM2 was upregulated in AP; its inhibition alleviated pancreatic injury and inflammation. Mechanistically, TGM2 bound STAT6 to suppress its phosphorylation/nuclear translocation, downregulating efferocytosis‐related GAS6 and impairing macrophage efferocytosis. LF‐LNP@si‐TGM2 targeted pancreatic macrophages, silenced TGM2, restored the STAT6–GAS6 axis, enhanced efferocytosis, and reduced inflammation.

**Conclusion:**

This study identifies TGM2 as a key regulator of AP macrophage efferocytosis via the novel TGM2–STAT6–GAS6 axis. LF‐LNP@si‐TGM2 is a promising targeted strategy for AP, potentially shifting treatment from supportive to precision therapy.

## Graphical abstract

This study shows TGM2 is upregulated in AP, impairing macrophage efferocytosis by inhibiting the STAT6–GAS6 axis. The lactoferrin‐modified, ROS‐responsive LF‐LNP@si‐TGM2 targets pancreatic macrophages, silences TGM2, restores the axis, and alleviates AP.

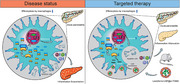

## 1 Background

Acute pancreatitis (AP) is one of the most common causes of acute abdomen worldwide. Approximately 80% of patients present with mild acute pancreatitis (Mild AP, MAP), which typically resolves within 1–2 weeks with supportive therapy. However, about 20% of patients progress to severe acute pancreatitis (Severe AP, SAP), characterized by persistent organ failure, pancreatic necrosis, and infectious complications, with a mortality rate reaching 20%–30% [1, 2]. Currently, the clinical management of AP remains largely supportive, focusing on fluid resuscitation, pain control, and infection management [3, 4]. However, effective targeted interventions addressing the core mechanisms underlying disease progression are still lacking. This limitation primarily stems from the fact that the molecular regulatory networks driving the transition from mild to severe AP have not yet been fully elucidated.

The pathobiology of AP is initiated by intra‐acinar premature activation of digestive enzymes (e.g., trypsinogen) triggered by etiologies such as gallstones, alcohol, and hypertriglyceridemia, leading to acinar cell injury and a cascade of local and systemic inflammation [5, 6]. Importantly, acinar cell death occurs along a spectrum, and necrosis—particularly in severe disease—represents the dominant driver of inflammatory amplification, as necrotic cells release damage‐associated molecular patterns (DAMPs) that robustly activate innate immunity [7]. In contrast, apoptosis is generally considered a more immunologically “silent” or even protective mode of cell death, because cellular contents remain compartmentalized and can be cleared before escalating inflammation [7, 8]. As DAMP burden increases, neutrophils and monocyte‐derived macrophages infiltrate the pancreas, fueling cytokine/chemokine release and promoting systemic inflammatory responses and organ dysfunction [7, 9]. Within this context, macrophages are not only cytokine producers but also professional scavengers responsible for clearing dying cells and debris [9, 10]. Efferocytosis—the phagocytic clearance of apoptotic cells—can limit secondary necrosis, reduce DAMP exposure, and facilitate a pro‐resolving macrophage program, thereby helping terminate inflammation rather than simply suppress it [10, 11]. Emerging evidence suggests that impaired clearance capacity and failure of resolution programs contribute to persistent inflammation, immune dysregulation, and infectious complications in severe AP [10, 11, 12].

Clinically, most patients with mild AP recover with standard supportive care (fluid resuscitation, analgesia, nutritional support, and complication management) [3, 5, 13]. The central unmet need is therefore not routine MAP treatment, but (i) preventing early progression in patients at high risk of developing SAP and (ii) mitigating the inflammatory–immune derailment that accompanies pancreatic necrosis and organ failure [5, 14]. Once extensive necrosis and infected necrosis are established, mortality is often driven by complications that may require procedural or surgical management; accordingly, any immunomodulatory strategy should be positioned as an adjunct and is most plausibly effective within defined windows (e.g., early disease or peri‐necrotic inflammation) rather than as a substitute for standard care [14, 15]. Prior attempts to broadly block single cytokines or inflammatory mediators have yielded inconsistent benefits, likely reflecting the heterogeneity and time‐dependent nature of the inflammatory microenvironment [12]. This motivates approaches that restore immune balance and facilitate resolution without indiscriminate immunosuppression [11, 12]. In this regard, targeting macrophage programs that couple pro‐inflammatory signaling with defective clearance/resolution is mechanistically attractive: improving the clearance of dying cells and inflammatory debris may reduce DAMP‐driven amplification, promote a transition toward pro‐resolving phenotypes, and thereby attenuate systemic immune dysregulation associated with severe disease [9, 10, 11]. To enable such cell‐directed modulation in vivo, lipid nanoparticles (LNPs) represent a mature non‐viral platform for siRNA delivery; their translational feasibility is supported by the successful clinical development and commercialization of siRNA–LNP therapeutics. By encapsulating siRNA, LNPs protect it from nuclease degradation, enhance cellular uptake, and facilitate endosomal escape for cytosolic gene silencing, while generally maintaining favorable biocompatibility and an acceptable safety profile [16, 17, 18].

This study first integrated bulk transcriptomic data and single‐cell RNA sequencing (scRNA‐seq) data from public databases, combined with machine learning algorithms, to identify core targets associated with the progression of AP to severe disease, ensuring both clinical relevance and specificity of the selected targets. Subsequently, in vitro cell models and in vivo AP mice models were used to verify the regulatory effects of the identified target on macrophage phenotypic transition, efferocytic function, and inflammatory balance, thereby elucidating its specific role in AP progression. Finally, an LNPs–siRNA delivery system targeting inflamed pancreatic tissue was constructed. By optimizing the lipid formulation, the system's macrophage‐targeting capability and siRNA release efficiency were enhanced. The therapeutic efficacy of this system was further validated in animal models, aiming to provide new molecular targets and technical strategies for precision‐targeted therapy of AP.

## 2 Methods

### 2.1 Materials

DSPC (000110), cholesterol (000123), DSPE‐TK‐PEG2000‐COOH (TK‐100501), ALC‐0315 (000117), and ALC‐0159 (029104) were purchased from MeloPEG (China). Cystamine (HY‐124476), AS1517499 (HY‐100614), Batiraxcept (HY‐P99463), and caerulein (HY‐A0190) were obtained from Med Chem Express (MCE, USA). pHrodo Red succinimidyl ester (P36600) was purchased from Thermo Fisher Scientific.

### 2.2 Cell Culture and Treatment

The mice macrophage cell line RAW 264.7 (CL‐0190, RRID: CVCL_0493) and acinar cell line 266‐6 (CL‐0974, RRID: CVCL_3481) were purchased from Wuhan Procell (Wuhan, China) in December 2023. The cell lines used in this study have been authenticated by STR profiling within the last three years. 266‐6 cells were cultured in RPMI‐1640 medium supplemented with 10% fetal bovine serum (FBS), 1% penicillin–streptomycin, 1% non‐essential amino acids (NEAA), and 1 mmol/L sodium pyruvate at 37°C in a humidified incubator containing 5% CO_2_ and ≥95% humidity. The culture medium was replaced every two days, and cells were passaged when 70%–80% confluent. For modeling, 266‐6 cells were serum‐starved for 4 h and then treated with 100 nm caerulein for 24 h. RAW264.7 cells were maintained in DMEM containing 10% FBS and 1% penicillin–streptomycin under the same culture conditions. The medium was changed every two days, and cells were passaged when 80%–90% confluent. For treatment, RAW264.7 cells were preincubated with 100 nm AS1517499 for 30 min, followed by stimulation with supernatants collected from differently treated 266‐6 cells for 24 h.

### 2.3 Animal Housing and Treatment

All animal experiments were approved by the Ethics Committee of the First Affiliated Hospital of Harbin Medical University (Approval No. YS447). Specific‐pathogen‐free (SPF) grade male C57BL/6 mice (8 weeks old, weighing 20–25 g) were acclimated for 1 week before the start of the experiment. Before inducing AP, the mice were pretreated as follows: cystamine for TGM2 inhibition (225 mg/kg, intraperitoneal injection, once daily for 7 consecutive days), Batiraxcept for GAS6 inhibition (100 mg/kg, intraperitoneal injection, once every 2 days, totaling 3 doses), AS1517499 for STAT6 phosphorylation inhibition (10 mg/kg, intraperitoneal injection, administered once 1 h before AP induction), and Lactoferrin‐Modified siTGM2‐Loaded Lipid Nanoparticles (LF‐LNP@si‐TGM2, 2 mg/kg, tail vein injection, administered once 24 h before model establishment).

The AP model was established by intraperitoneal injection of caerulein (50 µg/kg) every hour, with a total of 12 injections. Mice in the control group were given an equal volume of normal saline. Twenty‐four hours after the last injection, the mice were anesthetized and sacrificed, and peripheral blood and pancreatic tissues were collected for subsequent analysis. To mimic AP resembling severe gallstone‐induced acute pancreatitis, we established a SAP model via pancreatic duct ligation (PDL), as described in previous studies [19]. The detailed protocol is as follows: 6–8‐week‐old male C57BL/6J mice were selected and fasted overnight prior to the experiment, followed by anesthesia with CO_2_. After anesthesia, a midline abdominal incision was made, the duodenum was gently exteriorized, and the pancreatic duct was identified and ligated. Finally, the incision was sutured layer by layer with 8‐0 sutures. At 48 h post‐ligation, the mice received intraperitoneal injections of caerulein at a dose of 50 µg/kg body weight, with two injections administered at 1‐hour intervals. Simultaneously, the first administration of different formulations was performed on the mice; subsequent administrations of these formulations were also given within the two days following modeling. On the second day after the final administration, the mice were sacrificed, and blood samples as well as pancreatic, hepatic, and lung tissues were collected for subsequent detection and analysis.

### 2.4 Hematoxylin and Eosin Staining

Tissue samples from mice were fixed in 4% paraformaldehyde solution, followed by graded ethanol dehydration and embedding in paraffin blocks. The paraffin‐embedded tissues were sectioned into continuous 4‐µm‐thick slices using a Leica RM2135 rotary microtome. The sections were then stained with hematoxylin and eosin (H&E) and examined under a light microscope to evaluate histopathological and morphological changes in the pancreatic tissues.

### 2.5 Immunohistochemistry

Pancreatic tissues were fixed overnight in 4% paraformaldehyde, dehydrated, embedded in paraffin, and sectioned into 4‐µm‐thick slices. Antigen retrieval was performed using Tris‐EDTA buffer (pH 8.0) under high pressure for 2 min. Endogenous peroxidase activity was blocked with 3% hydrogen peroxide, followed by blocking with 10% goat serum at 37°C for 1 h. The sections were incubated overnight at 4°C with the following primary antibodies: TGM2 (Proteintech, 15100‐1‐AP, 1:100), IL‐1β (Affinity, AF5103, 1:100), IL‐6 (Affinity, DF6087, 1:100), and TNF‐α (Affinity, AF7014, 1:100). After washing, the sections were incubated with HRP‐conjugated secondary antibodies at 37°C for 30 min, followed by DAB chromogenic development for 5–10 min. Images were captured under a light microscope, and quantitative analysis was performed using ImageJ software.

### 2.6 Immunofluorescence (IF)

For tissue sections, antigen retrieval was performed in citrate buffer using a microwave oven, followed by blocking with 5% bovine serum albumin (BSA) for 1 h. Sections were then incubated with primary antibodies overnight at 4°C, washed three times with PBS, incubated with fluorescently labeled secondary antibodies, and counterstained with DAPI for nuclear staining. For cultured cells, samples were fixed with paraformaldehyde, blocked with 5% BSA for 1 h, incubated with primary antibodies overnight at 4°C, washed three times with PBS, followed by incubation with fluorescent secondary antibodies and DAPI staining. The following primary antibodies were used: TGM2 (Proteintech, 15100‐1‐AP, 1:100), IL‐1β (Affinity, AF5103, 1:100), IL‐6 (Affinity, DF6087, 1:100), TNF‐α (Affinity, AF7014, 1:100), CD86 (Huabio, ER1906‐01, 1:100), CD206 (CST, #24595, 1:100), F4/80 (Abcam, ab300421, 1:100), and Ly6G (Abcam, ab218132, 1:100).

### 2.7 Western Blot

Total proteins were extracted from pancreatic tissues or macrophages using RIPA lysis buffer, and the protein concentrations were determined by the BCA method. After separation by 8%–12% SDS‐PAGE, proteins were transferred onto PVDF membranes. The membranes were blocked with 5% skim milk at room temperature for 1 h. Primary antibodies—TGM2 (Proteintech, 15100‐1‐AP, 1:20000), GAS6 (Proteintech, 13795‐1‐AP, 1:1000), STAT6 (CST, #5397, 1:1000), p‐STAT6 (CST, #56554, 1:1000), p‐JAK1 (CST, #3331, 1:1000) and p‐JAK3 (Proteintech, 29101‐1‐AP, 1:1000)—were incubated overnight at 4°C. After washing with PBST, fluorescent secondary antibodies (LI‐COR) were added and incubated at room temperature for 1 h, followed by additional washing. Protein bands were visualized and quantified using the Odyssey CLx imaging system, with GAPDH serving as an internal control, and analyzed with ImageJ software.

### 2.8 Enzyme‐Linked Immunosorbent Assay

Blood samples were collected from the retro‐orbital venous plexus and centrifuged at 3000 rpm for 15 min at 4°C to obtain serum. Levels of IL‐1β (JonlnBio, JL18442), IL‐6 (JonlnBio, JL20268), and TNF‐α (JonlnBio, JL10484) were measured using Enzyme‐Linked Immunosorbent Assay (ELISA) kits according to the manufacturer's instructions. Serum amylase activity was determined using a Solarbio amylase assay kit (BC5055) following the provided protocol. Mouse TNNT2 (BP02712), ALT (BP10018W), AST (BP10019W), Cr (BP10020W), and BUN (BP10383W) ELISA kits were purchased from Bepeng Biotech Co., Ltd. (Shanghai, China).

### 2.9 Flow Cytometry

Cells were collected and centrifuged at 5000 × g for 5 min, then resuspended in PBS. Fluorescence intensity was measured using a FACS Calibur flow cytometer with detection in the FL1 channel for FITC fluorescence. The mean fluorescence intensity (MFI) of each group was calculated to compare the cellular uptake efficiency.

Pancreatic immune cells were isolated via the Collagenase P digestion method and then passed through a 70 µm cell strainer for separation. After washing and resuspending the cells, they were surface‐stained with CD45 (Elabscience, E‐AB‐F1136D), CD11b (Elabscience, E‐AB‐F1081E), F4/80 (Elabscience, E‐AB‐F0995J), Ly6G (Elabscience, E‐AB‐F1108H), and TGM2 (Proteintech, CL488‐68006) antibodies at 4°C for 60 min. Finally, the stained cells were detected and analyzed on a NovoCyte flow cytometer (RaiseCyte, RaiseCyte 2L6C).

### 2.10 Quantitative Real‐Time Polymerase Chain Reaction

Total RNA was extracted from RAW264.7 cells using TRIzol reagent (Invitrogen, USA). According to the manufacturer's instructions, purified RNA was reverse‐transcribed into complementary DNA (cDNA) using the Transcriptor First Strand cDNA Synthesis Kit (Roche, Germany). Quantitative PCR was performed with SYBR Green (Roche, Germany) on a CFX Connect Real‐Time PCR Detection System (Bio‐Rad, USA). Details of the primer sequences used for qPCR are provided in Table S1. Relative gene expression levels were calculated using the 2^−^Δ(ΔCt) method.

### 2.11 Cell Transfection

Small interfering RNAs (siRNAs) targeting TGM2 and GAS6, as well as the plasmid pcDNA3.1‐STAT6 (Tyr641Phe), were purchased from GenePharma Co., Ltd. (Shanghai, China). RAW264.7 cells were seeded into 6‐well plates at a density of 2 × 10^5^ cells per well. Transfection was performed with 50 nm siRNA using a transfection reagent (Invitrogen, California, USA) according to the manufacturer's instructions. Knockdown efficiency was verified by qPCR and Western blotting. The sequences of the siRNAs and pcDNA3.1‐STAT6 used in this study are listed in Table S2.

### 2.12 Co‐Immunoprecipitation

Protein samples were incubated overnight at 4°C on a rotating shaker with IP antibodies against the following targets: TGM2 (Proteintech, 15100‐1‐AP, 1:50), STAT6 (Cell Signaling Technology, #5397, 1:50), and IgG (Abcam, UK, 1:50). Protein A/G magnetic beads (Bimake, B23202, USA) were washed three times with RIPA buffer and then blocked with 5% BSA at 4°C for 30 min. The protein–antibody mixture was subsequently incubated with the Protein A/G beads at 4°C for 6 h to allow binding. After incubation, the supernatant was discarded, and 100 µL of lysis buffer (1 × SDS loading buffer) was added to elute the bound proteins. The samples were then boiled for 5 min and analyzed by SDS‐PAGE.

### 2.13 Chromatin Immunoprecipitation Followed by Quantitative Real‐Time PCR

Samples were cross‐linked with 1% formaldehyde at room temperature, and the reaction was quenched with glycine. After centrifugation, the cells were washed, lysed, and subjected to ultrasonic shearing using the working lysis buffer and ChIP buffer provided in the Bersinbio ChIP Kit (bes5001, China). Cross‐linked DNA was immunoprecipitated using a ChIP‐grade anti‐STAT6 antibody (Cell Signaling Technology, #5397) or IgG as a nonspecific control. The immune complexes were captured using Protein A/G magnetic beads, followed by reverse cross‐linking and DNA purification for subsequent qPCR analysis. The primer sequences used for ChIP–qPCR in this study are listed in Table S3.

### 2.14 Dual‐Luciferase Reporter Assay

The GAS6 promoter wild‐type (pGLO‐GAS6‐Promoter‐WT) and STAT6 binding site mutant (pGLO‐GAS6‐Promoter‐MUT) reporter plasmids were constructed and co‐transfected into RAW264.7 cells with either pcDNA3.1‐STAT6 or an empty vector. After 24 h, the cells were lysed and centrifuged at 12 000 rpm for 1 min to collect the supernatant. The activities of firefly and Renilla luciferases were measured using the Dual‐Luciferase Reporter Assay System (Promega, USA). The ratio of firefly to Renilla luciferase activity was used to evaluate the transcriptional activation ability of STAT6 on the GAS6 promoter.

### 2.15 Synthesis of LF‐LNP@si‐TGM2

LF‐LNP@si‐TGM2 nanoparticles were prepared via a microfluidic mixing technique (Model: FluidicLab LNP‐B1; flow rate ratio: 1:3; total flow rate: 12 mL/min; mixing temperature: room temperature). The detailed preparation procedure is described as follows. DSPE‐TK‐PEG2000‐COOH, ALC‐0315, ALC‐0159, DSPC, and cholesterol were dissolved in ethanol at a molar ratio of 0.5:50:1:10:38.5. The si‐TGM2 was dissolved in 100 mm citrate buffer (pH 4–6). The aqueous siRNA solution and ethanol lipid solution were rapidly mixed at a volume ratio of 3:1 and incubated at room temperature to allow the self‐assembly of LNPs. The resulting LNPs were dialyzed in sterile PBS for 2 h to remove ethanol. Next, 4 mg of NHS and 6 mg of EDC were added to the LNP suspension for activation at 4°C for 2 h, followed by the addition of 5 mg of lactoferrin. The reaction was continued overnight at 4°C. Unconjugated lactoferrin was removed by filtration, and the final product was purified by dialysis in PBS for 2 h.

### 2.16 In Vitro Hemolysis Assay

To evaluate the blood compatibility of the nanosystems, whole blood was collected from freshly euthanized mice and centrifuged at 3000 rpm for 5 min to isolate red blood cells (RBCs). After discarding the plasma, the RBCs were washed with PBS (10 mM, pH 7.4) and resuspended to a 2% RBC suspension. LF‐LNP@si‐TGM2 were separately added to the 2% RBC suspension to achieve final concentrations of 10, 50, 200, 500, and 1000 µg/mL, respectively. PBS and distilled water were used as the negative control and positive control, respectively. All samples were incubated at 37°C for 3 h, then centrifuged at 1500 rpm for 10 min, and the supernatants were collected. The absorbance of each group was measured at a wavelength of 540 nm using a microplate reader, and the hemolysis rate was calculated according to the following formula:

$$\;\mathrm{Hemolysis}\;(\%)=\frac{A_{\mathrm{sample}}-A_{\mathrm{negative}}}{A_{\mathrm{positive}}-A_{\mathrm{negative}}}\;\times 100\%$$

where *A_sample_*, *A_negative,_* and *A_positive_* represent the absorbance values of the experimental group, negative control group, and positive control group, respectively.

### 2.17 Efferocytosis Assay

For the in vitro efferocytosis assay, apoptotic 266‐6 acinar cells (ACs) were induced by treatment with an excess of caerulein, followed by labeling with 1 µg/mL pHrodo Red succinimidyl ester (Thermo Fisher Scientific). Macrophages were pretreated with si‐TGM2 or LF‐LNP@si‐TGM2, and then co‐cultured with pHrodo Red‐labeled ACs. After incubation at 37°C for 1 h, the cells were washed with PBS to remove unphagocytosed ACs and stained with FITC‐conjugated anti‐F4/80 antibody to identify macrophages. Fluorescence images were captured using a fluorescence microscope, and the red fluorescence intensity represented the degree of efferocytosis. For the in vivo efferocytosis assay, two complementary methods were applied. First, transmission electron microscopy (TEM) was used to directly visualize macrophage‐mediated engulfment of apoptotic cells. Second, apoptotic cells in pancreatic tissues were labeled using a TUNEL apoptosis detection kit (Beyotime, China), while macrophages were stained with FITC‐conjugated anti‐F4/80 antibody. Fluorescent co‐localization was observed under a fluorescence microscope to assess in situ efferocytic activity.

### 2.18 Bioinformatics Analysis

Bulk RNA‐seq datasets GSE109227 (6 AP vs. 4 NC) and GSE65146 (9 AP vs. 6 NC) were obtained from GEO database (https://www.ncbi.nlm.nih.gov/geo/), merged in R using ComBat to remove batch effects, and AP signature genes were selected with SVM‐RFE (e1071 package) and random forest (randomForest package). Single‐cell RNA sequencing datasets GSE188819 (1 AP vs. 1 NC), GSE198183 (1 AP vs. 1 NC), and GSE181276 (1 AP) were analyzed using the Seurat pipeline. Cells with 200–6000 detected genes and <10% mitochondrial gene expression were retained; batch effects were corrected with harmony; FindVariableFeatures was used to select the top 3000 variable genes; and cell subtypes were annotated with reference to the CellMarker 2.0 database. Gene Ontology (GO) and Kyoto Encyclopedia of Genes and Genomes (KEGG) enrichment analyses were performed using the clusterProfiler package (*p* < 0.05 and adjusted *p* < 0.05). Protein–protein interaction (PPI) networks were constructed using GeneMANIA (http://genemania.org/) and STRING (https://string‐db.org/). ZDOCK was employed for TGM2–STAT6 protein docking; after structural preprocessing, calculations were run with default parameters. The top‐scoring poses were further screened with molecular dynamics, and interaction sites and stability were evaluated.

For Mendelian Randomization (MR) analysis, the protein quantitative trait locus (pQTL) data was derived from the study by Karsten Suhre et al. [20], which included the expression level measurements of 1463 proteins from 54 000 participants. The genome‐wide association study (GWAS) data for the discovery set of AP was obtained from the ieu OpenGWAS database (https://opengwas.io/) with the accession number ebi‐a‐GCST90018789. In this dataset, the case group consisted of 3798 patients with confirmed AP, while the control group included 4 76 104 healthy participants without a history of AP.​The GWAS data for the validation set of AP was retrieved from the R11 version of the FinnGen database (accession path: gs://finngen‐public‐data‐r11/summary_stats/finngen_R11_K11_ACUTPANC.gz). Here, the case group comprised 7562 patients with confirmed AP, and the control group included 3 97 583 healthy participants without a history of AP.​For the aforementioned 1463 proteins, the pQTL loci corresponding to each protein were used as potential instrumental variables (IVs), with the following screening criteria: (1) The genetic variants showed a significant association with the exposure factor (protein expression level), with a set threshold of *p* < 5 × 10^−^^6^; (2) Genetic variants with Linkage Disequilibrium (LD) were excluded. Using the European population as the reference panel (e.g., 1000 Genomes Project), the LD coefficient r^2^ was set to < 0.001 and the genetic distance to > 10 000 kb. Ultimately, analyzable IVs were obtained for 1001 proteins, which were used for subsequent MR analysis. The Inverse Variance Weighted method was adopted as the primary analytical approach to calculate the causal effect size (Odds Ratio, OR) and 95% Confidence Interval (CI) between each protein and the risk of AP, thereby exploring the potential causal associations between these 1001 proteins and AP. A false discovery rate (FDR) < 0.05 was set as the threshold to determine statistical significance.

### 2.19 Statistical Analysis

Continuous variables were presented as mean ± standard deviation (SD). Depending on the normality of the data distribution, either the t‐test or Mann–Whitney U test was used to compare differences between the two groups. Spearman's rank correlation analysis was performed for correlation assessment. All statistical analyses were conducted using GraphPad Prism software. A value of *p* < 0.05 was considered statistically significant (significance markers: ^*^ *p* < 0.05, ^**^ *p* < 0.01, ^***^ *p* < 0.001).

## 3 Results

### 3.1 Identification of TGM2 as a Core Target in AP

**FIGURE 1:**
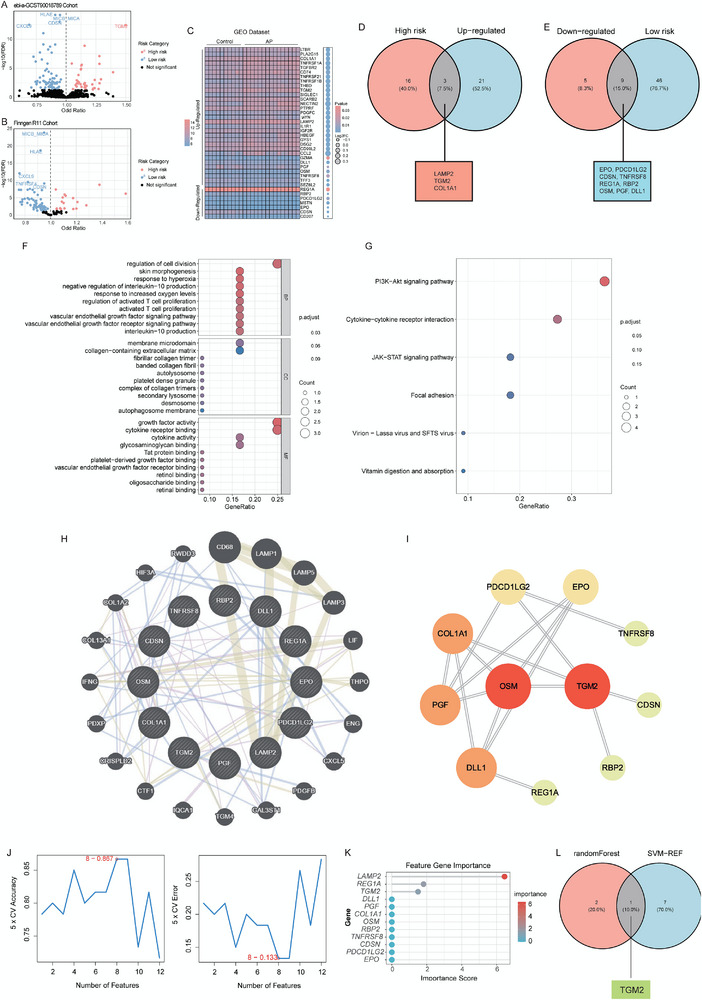
Screening and identification of TGM2 as a key target in AP. (A) Mendelian randomization (MR) analysis showing the distribution of 105 proteins associated with AP risk in the ebi‐a‐GCST90018789 dataset. Red dots represent proteins positively associated with higher AP risk (36 proteins), while blue dots represent proteins associated with lower AP risk (69 proteins). (B) Validation of the ebi‐a‐GCST90018789 dataset in the Finngen cohort. The volcano plot shows 73 proteins that remain significantly associated with AP risk. (C) Heatmap showing differential expression of 60 AP risk‐related protein‐coding mRNAs in the AP transcriptomic dataset. (D) Intersection of MR‐identified high‐risk proteins with upregulated mRNAs from transcriptomic analysis. (E) Intersection of MR‐identified low‐risk proteins with downregulated mRNAs. (F,G) GO and KEGG enrichment bubble plots illustrating biological processes and signaling pathways associated with the 12 overlapping molecules. (H) GeneMANIA database and (I) STRING database analyses constructing the protein–protein interaction (PPI) network of the 12 key proteins. (J,K) Importance ranking of candidate genes evaluated by two machine learning algorithms: SVM‐REF and Random Forest. (L) Both machine learning models identified TGM2 as a key gene in AP. ^*^ *p* < 0.05, ^**^ *p* < 0.01, ^***^ *p* < 0.001.

In the ebi‐a‐GCST90018789 dataset, a total of 105 proteins were identified to be significantly associated with the risk of AP (FDR < 0.05). Among them, 69 proteins were correlated with a lower AP risk, while 36 proteins were associated with a higher AP risk (Figure 1A). Further validation using the FinnGen dataset confirmed that 73 proteins remained significantly associated with AP risk (FDR < 0.05, Figure 1B). Differential expression analysis of the mRNAs encoding these 73 proteins in AP transcriptomic datasets revealed that transcriptional data were unavailable for 14 proteins, whereas 38 out of the remaining 60 proteins showed significant expression dysregulation, including 24 upregulated and 14 downregulated mRNAs (*p* < 0.05, Figure 1C). Specifically, among the high‐risk‐associated proteins, the mRNA levels of LAMP2, TGM2, and COL1A1 were significantly elevated in pancreatic tissues of AP patients (Figure 1D). Conversely, among the low‐risk‐associated proteins, the mRNA levels of EPO, PDCD1LG2, CDSN, TNFRSF8, REG1A, RBP2, OSM, PGF, and DLL1 were significantly decreased (Figure 1E). GO enrichment analysis revealed that the 12 identified molecules were mainly involved in biological processes such as response to hyperoxia, negative regulation of interleukin‐10 production, and regulation of activated T cell proliferation (Figure 1F). KEGG pathway enrichment analysis further showed that these molecules were predominantly enriched in the PI3K–Akt signaling pathway, cytokine–cytokine receptor interaction, and JAK–STAT signaling pathway (Figure 1G). In PPI analyses, the GeneMANIA database identified the top 20 proteins interacting with the 12 candidate proteins, forming a highly connected network (Figure 1H). Meanwhile, STRING database analysis indicated that LAMP2 did not form any detectable interactions, whereas OSM and TGM2 occupied the central nodes within the interaction network (Figure 1I), suggesting that TGM2 may play a pivotal regulatory role in the AP‐related signaling network.

To prioritize core molecules in AP, we evaluated the importance of the 12 candidate mRNAs using two machine‐learning algorithms. SVM‐RFE analysis indicated that the model achieved the highest accuracy (0.867, Figure 1J) when eight mRNAs—RBP2, TGM2, COL1A1, EPO, PGF, DLL1, TNFRSF8, and LAMP2—were included. In parallel, random forest ranking showed that the importance scores of LAMP2, REG1A, and TGM2 were > 1 (Figure 1K). Notably, TGM2 was identified as an AP signature gene by both models (Figure 1L). Immune infiltration analysis based on transcriptome data showed that in pancreatic tissues, the expression of TGM2 was positively correlated with the infiltration scores of monocytes and M1 macrophages, while negatively correlated with the infiltration scores of CD8+ T cells and Treg cells. This suggests that TGM2 may be associated with pro‐inflammatory properties (Figure S1). Collectively, these multi‐omics analyses nominate TGM2 as a candidate AP‐associated regulator and prioritize it for downstream mechanistic and therapeutic validation.

### 3.2 TGM2 is Up‐Regulated in AP and Promotes Disease Progression

**FIGURE 2:**
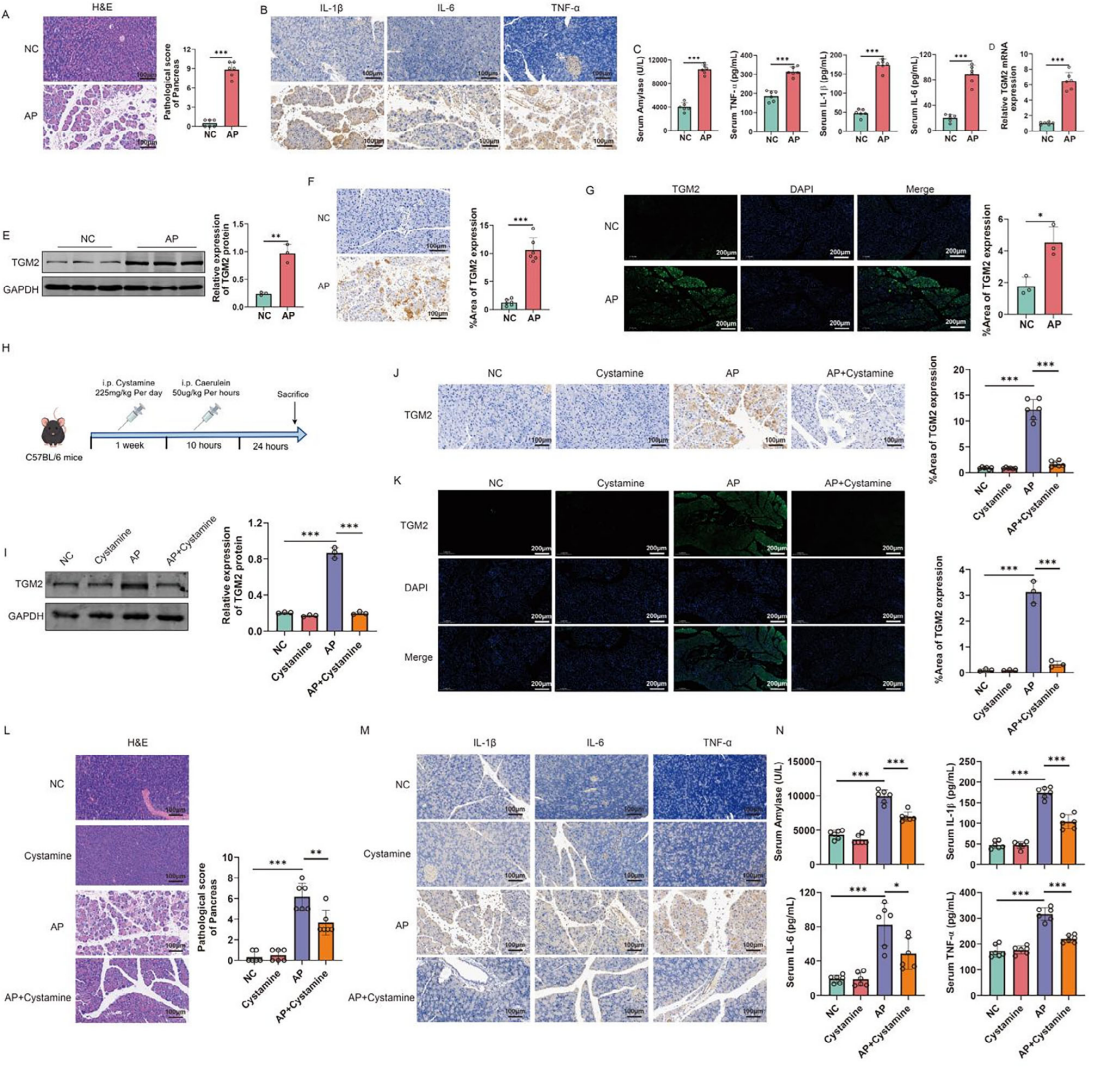
Upregulation of TGM2 in AP and its role in promoting disease progression. (A) Comparison of pancreatic tissue morphology between normal control (NC) and AP model mice by hematoxylin–eosin (H&E) staining (n = 6). (B) Immunohistochemical (IHC) staining of IL‐1β, IL‐6, and TNF‐α in pancreatic tissues from NC and AP mice (n = 6). (C) ELISA results showing serum amylase activity and the levels of IL‐1β, IL‐6, and TNF‐α in peripheral blood of NC and AP mice (n = 6). (D–G) Quantification of TGM2 expression in pancreatic tissues from NC and AP mice by qPCR (D, n = 6), Western blot (E, n = 3), IHC (F, n = 6), and immunofluorescence (IF) staining (G, n = 3). (H) Schematic illustration of the experimental design showing the pretreatment protocol with the TGM2 inhibitor Cystamine prior to AP induction. (I–K) Western blot (I, n = 3), IHC (J, n = 6), and IF (K, n = 3) results demonstrating that Cystamine effectively inhibits TGM2 expression in pancreatic tissues. (L) H&E staining showing that Cystamine pretreatment alleviates pancreatic damage in AP mice (n = 6). (M) IHC staining indicating that Cystamine pretreatment reduces IL‐1β, IL‐6, and TNF‐α expression in pancreatic tissues (n = 6). (N) ELISA results showing that Cystamine pretreatment decreases serum amylase activity and levels of IL‐1β, IL‐6, and TNF‐α in AP mice (n = 6). ^*^ *p* < 0.05, ^**^ *p* < 0.01, ^***^ *p* < 0.001.

To investigate the role of TGM2 in AP, we first established an AP mice model. Compared with NC mice, AP mice exhibited marked pancreatic injury, with significantly increased expression of IL‐1β, IL‐6, and TNF‐α in pancreatic tissues (Figure 2A,B). Similarly, serum amylase activity and circulating levels of IL‐1β, IL‐6, and TNF‐α were also markedly elevated (Figure 2C). qPCR, Western blot, and IHC analyses consistently demonstrated that TGM2 expression in the pancreatic tissues of AP mice was significantly higher than that in NC mice (Figure 2D–F). IF further confirmed that TGM2 was strongly upregulated in the pancreatic tissues of AP mice (Figure 2G). In addition, we established a SAP model to verify the expression of TGM2 (Figure S2A–F). qPCR, Western blot, and IHC results showed that compared with the control group, the expression level of TGM2 in pancreatic tissue of mice in the SAP group was significantly increased (Figure S2G–I). To evaluate the functional role of TGM2, we pretreated AP mice with the TGM2 inhibitor cystamine prior to disease induction (Figure 2H). Western blot, IHC, and IF results showed that cystamine effectively suppressed TGM2 expression in pancreatic tissues (Figure 2I–K). Histopathological staining results showed that cystamine pretreatment had no effect on the pancreatic tissue of normal mice, but significantly alleviate pancreatic injury and reduce the expression levels of IL‐1β, IL‐6, and TNF‐α in mice with AP (Figure 2L,M). Moreover, cystamine pretreatment also decreased serum amylase activity and the systemic levels of inflammatory cytokines in mice with AP (Figure 2N). Likewise, cystamine pretreatment alleviated the levels of pancreatic and systemic inflammation in SAP mice (Figure S3A–D). Taken together, these results indicate that TGM2 contributes to both local and systemic inflammatory responses in AP mice, supporting that TGM2 contributes to pancreatic injury and amplifies local and systemic inflammatory responses in experimental AP.

### 3.3 TGM2 Promotes Macrophage Inflammation in AP

**FIGURE 3:**
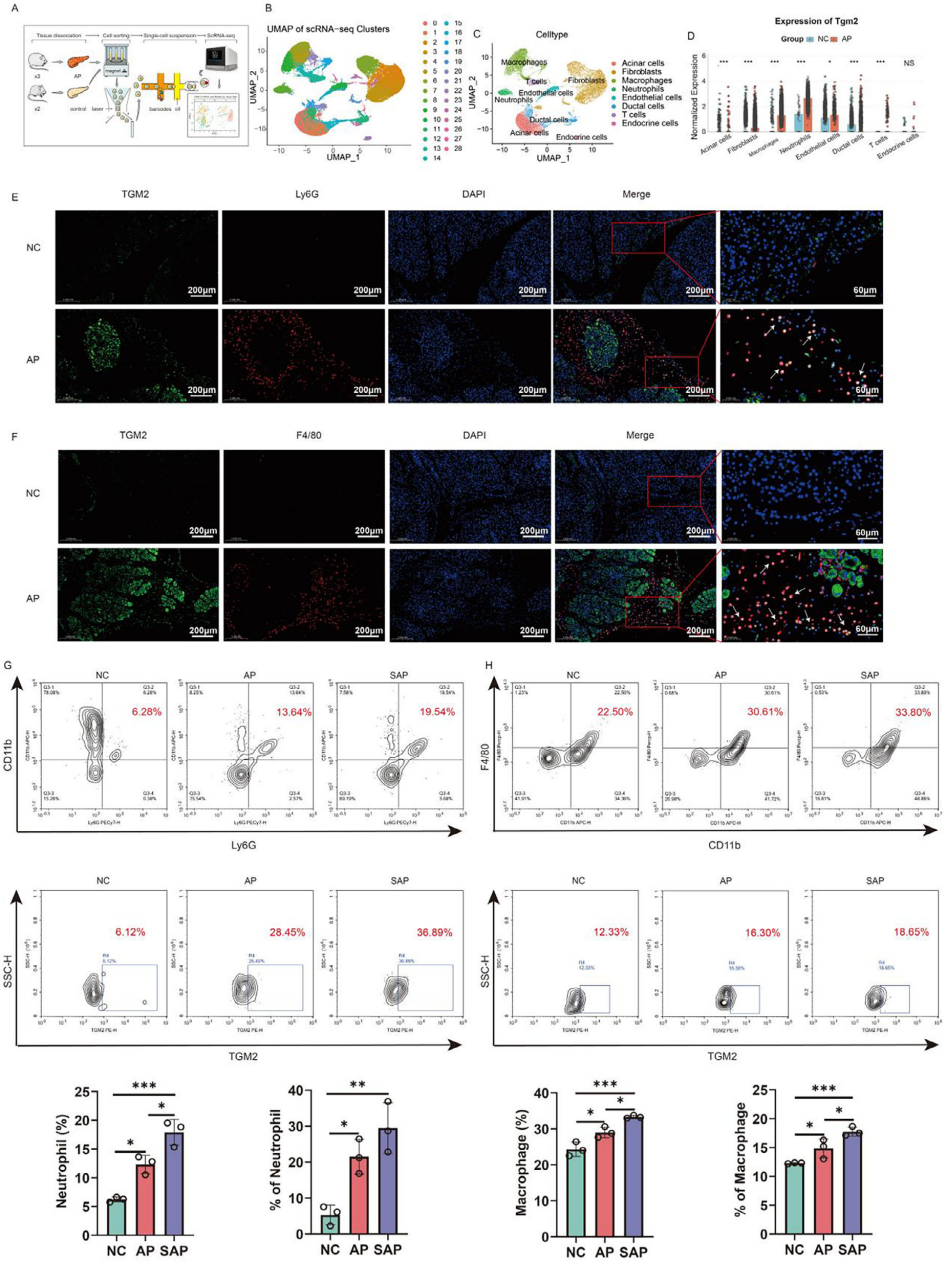
Expression pattern and pathway analysis of TGM2 in pancreatic cell subtypes of AP mice. (A) Schematic workflow of single‐cell RNA sequencing (scRNA‐seq) analysis. (B) Cell clustering of pancreatic tissues from normal control (NC) and AP mice based on scRNA‐seq data. (C) Cell type annotation results of identified clusters. (D) Comparison of TGM2 expression across different cell types in NC and AP groups. (E) Immunofluorescence (IF) co‐staining of Ly6G (a neutrophil marker, green) and TGM2 (red) in pancreatic tissues of NC and AP mice (n = 3). (F) IF co‐staining of F4/80 (a macrophage marker, green) and TGM2 (red) in pancreatic tissues of NC and AP mice (n = 3). (G) Flow cytometry determined the proportions of neutrophils and TGM2^+^ neutrophils in the pancreatic tissues of mice in the NC, AP, and SAP groups (n = 3). (H) Flow cytometry determined the proportions of macrophages and TGM2^+^ macrophages in the pancreatic tissues of mice in the NC, AP, and SAP groups (n = 3). ^*^ *p* < 0.05, ^**^ *p* < 0.01, ^***^ *p* < 0.001.

To explore the potential mechanism by which TGM2 aggravates AP, we analyzed publicly available scRNA‐seq datasets (Figure 3A; Figure S4A). A total of 29 cell clusters were identified from the pancreatic tissues of NC and AP mice (Figure 3B) and annotated into eight major cell types: acinar cells, fibroblasts, macrophages, neutrophils, endothelial cells, ductal cells, T cells, and endocrine cells (Figure 3C; Figure S4B). Compared with the NC group, the AP group exhibited a decreased proportion of acinar and endothelial cells, but an increased infiltration of neutrophils and macrophages (Figure S4C). TGM2 expression was primarily localized to neutrophils and macrophages, with significantly higher expression levels in AP tissues than in NC tissues (Figure 3D). We further divided both neutrophils and macrophages into TGM2^+^ and TGM2^−^ subpopulations for differential analysis (Figure S4D–G). In neutrophils, GO enrichment analysis revealed that the differentially expressed genes were enriched in biological processes related to positive regulation of cytokine production, leukocyte migration, myeloid cell differentiation, and myeloid leukocyte migration (Figure S4H). KEGG analysis showed enrichment in the IL‐17, TNF‐α, and NF‐κB signaling pathways (Figure S4I). In macrophages, GO analysis demonstrated enrichment in leukocyte migration and chemokine‐mediated signaling pathways (Figure S4J), while KEGG analysis identified involvement in efferocytosis, cytokine–cytokine receptor interaction, IL‐17 signaling, and PI3K–Akt signaling pathways (Figure S4K). Cell–cell communication analysis further revealed distinct interaction patterns between TGM2^+^ and TGM2^−^ neutrophils and macrophages, suggesting functional heterogeneity (Figures S5 and S6).

Subsequently, IF staining confirmed that both neutrophil (Ly6G^+^) and macrophage (F4/80^+^) infiltration were markedly increased in the pancreatic tissues of AP mice compared with NC mice, and that TGM2 was highly expressed in both cell types (Figure 3E,F). Finally, flow cytometry results demonstrated that, compared with the NC group, the proportions of neutrophils and macrophages in the pancreatic tissues of mice in the AP and SAP groups were significantly increased, along with elevated percentages of TGM2^+^ neutrophils and TGM2^+^ macrophages (Figure 3G,H).

**FIGURE 4:**
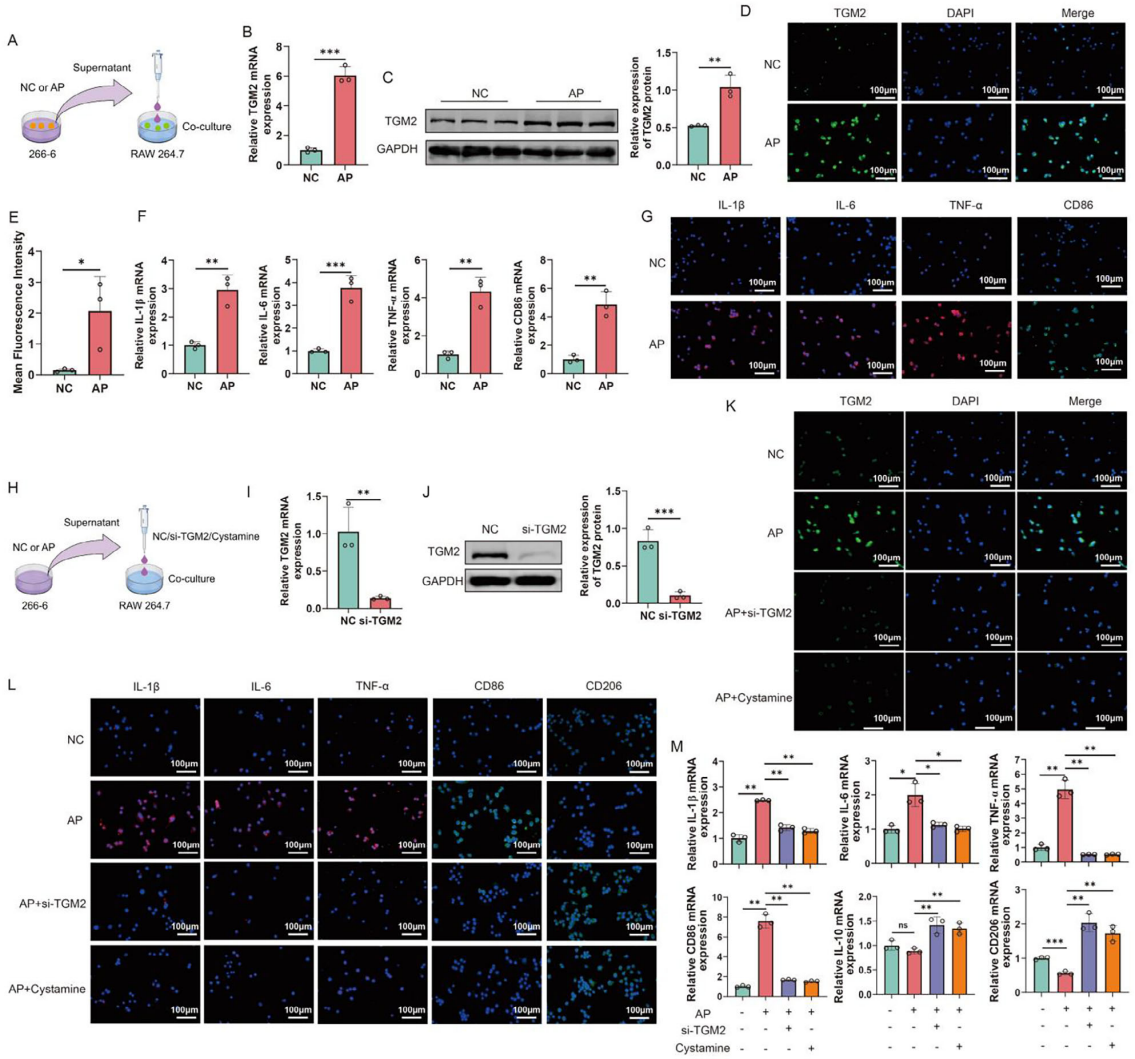
Regulatory role of TGM2 in macrophage inflammation under AP conditions. (A) Schematic diagram of the in vitro experimental design showing the treatment of RAW264.7 macrophages with supernatants from 266‐6 acinar cells in the AP group. (B–E) qPCR (B), Western blot (C), and immunofluorescence (IF) staining (D–E) results showing TGM2 expression in RAW264.7 macrophages treated with supernatants from normal control (NC) and AP 266‐6 cells (n = 3). (F,G) qPCR (F) and IF (G) analyses showing IL‐1β, IL‐6, and TNF‐α expression in RAW264.7 macrophages stimulated with NC or AP 266‐6 cell supernatants (n = 3). (H) Schematic diagram of the TGM2 knockdown experiment showing the transfection procedure of RAW264.7 cells with si‐TGM2. (I–K) qPCR (I), Western blot (J), and IF (K) results confirming the efficiency of TGM2 knockdown in RAW264.7 macrophages transfected with si‐NC (negative control) or si‐TGM2 (n = 3). (L,M) IF (L) and qPCR (M) results showing that TGM2 knockdown and inhibition significantly reduced the AP 266‐6 supernatant‐induced upregulation of IL‐1β, IL‐6, TNF‐α, and CD86, while increased IL‐10 and CD206 in RAW264.7 macrophages (n = 3). ^*^ *p* < 0.05, ^**^ *p* < 0.01, ^***^ *p* < 0.001.

To validate the above findings, we established an in vitro AP model using the mice acinar cell line 266‐6, and collected the supernatant to stimulate the macrophage cell line RAW264.7, thereby mimicking the AP microenvironment (Figure 4A). qPCR, Western blot, and IF analyses revealed that compared with the NC group, TGM2 mRNA and protein levels were significantly upregulated in RAW264.7 cells stimulated with AP supernatant (Figure 4B–E). Meanwhile, the expression of inflammatory cytokines IL‐1β, IL‐6, TNF‐α, and the M1 macrophage marker CD86 was markedly increased, indicating a shift toward pro‐inflammatory polarization (Figure 4F,G). To clarify the role of TGM2 in macrophage inflammatory responses, we performed TGM2 knockdown and cystamine inhibition experiments in RAW264.7 macrophages stimulated with AP supernatant (Figure 4H). qPCR, Western blot, and IF confirmed that TGM2 was efficiently silenced (Figure 4I–K). Further analysis demonstrated that TGM2 knockout and inhibition significantly reduced the high expression of IL‐1β, IL‐6, TNF‐α, and CD86 induced by the supernatant of 266‐6 cells from the AP group, while increasing the expression of the anti‐inflammatory cytokine IL‐10 and the M2 macrophage marker CD206 (Figure 4L,M). Taken together, these findings indicate that TGM2 promotes macrophage inflammatory responses and drives M1 polarization in the AP model.

### 3.4 TGM2 Aggravates AP by Inhibiting GAS6‐Dependent Macrophage Efferocytosis

**FIGURE 5:**
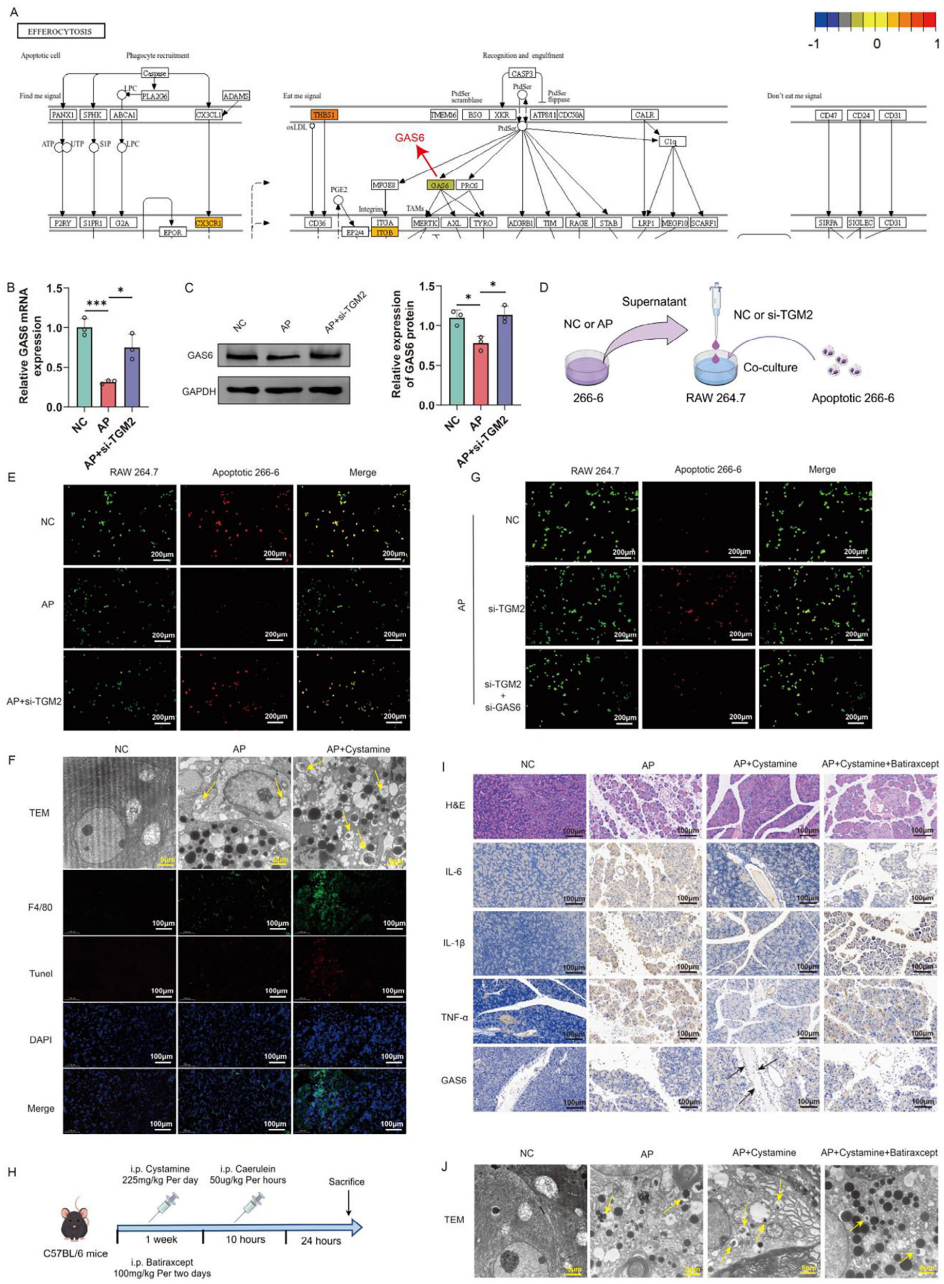
TGM2 aggravates AP by suppressing GAS6‐dependent macrophage efferocytosis. (A) Comparison of efferocytosis‐related gene expression (THBS1, ITGB, CX3CR1, and GAS6) between TGM2^+^ and TGM2^−^ macrophages. (B,C) qPCR (B) and Western blot (C) results showing that TGM2 knockdown significantly alleviated the reduction in GAS6 expression induced by AP 266‐6 cell supernatants in macrophages (n = 3). (D) Schematic illustration of the in vitro efferocytosis assay showing the co‐culture of apoptotic 266‐6 cells (ACs) with macrophages under different treatments. (E) TGM2 knockdown markedly restored the impaired phagocytic ability of macrophages toward ACs caused by stimulation with AP 266‐6 cell supernatants (n = 3). (F) Transmission electron microscopy (TEM) and immunofluorescence (IF) results showing that Cystamine pretreatment promoted efferocytosis in the pancreatic tissues of AP mice, and the area indicated by the arrow shows the scene where lysosomes phagocytose apoptotic cell debris (n = 3). (G) In macrophages stimulated with AP 266‐6 cell supernatants, GAS6 knockdown abrogated the TGM2 knockdown–mediated restoration of efferocytosis (n = 3). (H) Schematic diagram of the procedure for simultaneous inhibition of TGM2 and GAS6 in AP. (I) H&E staining and IHC staining showing that GAS6 inhibition impaired the protective effects of Cystamine pretreatment on pancreatic tissue injury and the reduction of IL‐1β, IL‐6, and TNF‐α expression in AP mice (n = 6). (J) TEM images showing that GAS6 inhibition attenuated the Cystamine pretreatment–induced enhancement of efferocytosis in pancreatic tissues of AP mice, and the area indicated by the arrow shows the scene where lysosomes phagocytose apoptotic cell debris (n = 3). ^*^ *p* < 0.05, ^**^ *p* < 0.01, ^***^ *p* < 0.001.

Given that KEGG enrichment analysis revealed significant differences in the efferocytosis pathway between TGM2^+^ and TGM2^−^ macrophages (Figure 3J), we further examined the expression patterns of efferocytosis‐related genes. The results showed that in TGM2^+^ macrophages, efferocytosis‐associated molecules such as THBS1, ITGB, and CX3CR1 were upregulated, whereas GAS6 expression was markedly downregulated (Figure 5A). Consistently, qPCR and Western blot analyses demonstrated that GAS6 expression was reduced in macrophages stimulated with AP‐conditioned medium derived from 266‐6 cells, while TGM2 knockdown significantly restored GAS6 expression (Figure 5B,C).

To evaluate macrophage efferocytosis, apoptotic 266‐6 cells were co‐cultured with macrophages subjected to different treatments (Figure 5D). The results showed that macrophages in the NC group efficiently engulfed apoptotic cells, exhibiting prominent efferocytosis. In contrast, macrophages stimulated with AP‐conditioned medium displayed significantly impaired efferocytic capacity. Notably, TGM2 knockdown reversed the AP‐induced suppression of efferocytosis (Figure 5E). In vivo, TEM and IF identified macrophages containing apoptotic material in pancreatic tissues of AP mice, indicating that efferocytic activity is present but may be insufficient to fully counterbalance the inflammatory milieu during pancreatitis (Figure 5F). Importantly, TGM2 inhibition further enhanced efferocytosis levels (Figure 5F).

To clarify the role of GAS6 in TGM2‐mediated regulation of efferocytosis, we simultaneously knocked down TGM2 and GAS6 in macrophages and stimulated the cells with AP‐conditioned medium. The results showed that GAS6 silencing reversed the improvement in efferocytic function caused by TGM2 knockdown (Figure 5G). In addition, GAS6 knockdown reversed the TGM2 knockdown‐induced downregulation of IL‐1β, IL‐6, TNF‐α, and CD86 expression, as well as the upregulation of CD206 and IL‐10 expression (Figure S7). Subsequently, we simultaneously inhibited TGM2 and GAS6 in AP mice (Figure 5H). IHC staining of pancreatic tissues showed that the expression level of GAS6 was low in both NC and AP mice, whereas TGM2 inhibition upregulated the expression level of GAS6 in pancreatic stromal cells of AP mice (Figure 5I). GAS6 inhibition reversed the protective effects of TGM2 inhibition, including the reduction of pancreatic injury and the downregulation of inflammatory cytokines (Figure 5I). Moreover, GAS6 inhibition attenuated the enhanced efferocytosis observed after TGM2 inhibition (Figure 5J). Likewise, in the SAP mice model, TGM2 inhibition alleviated pancreatic tissue damage and inflammation (Figure S8A) and enhanced efferocytosis levels (Figure S8B), while this effect was abolished following GAS6 inhibition (Figure S8). Taken together, these findings indicate that TGM2 aggravates the progression of AP by downregulating GAS6 expression, thereby impairing macrophage efferocytic function and potentially perpetuating inflammation, consistent with the notion that defective clearance can increase secondary necrosis–associated inflammatory spillover.

### 3.5 TGM2 Downregulates GAS6 Transcription by Inhibiting STAT6 Phosphorylation

**FIGURE 6:**
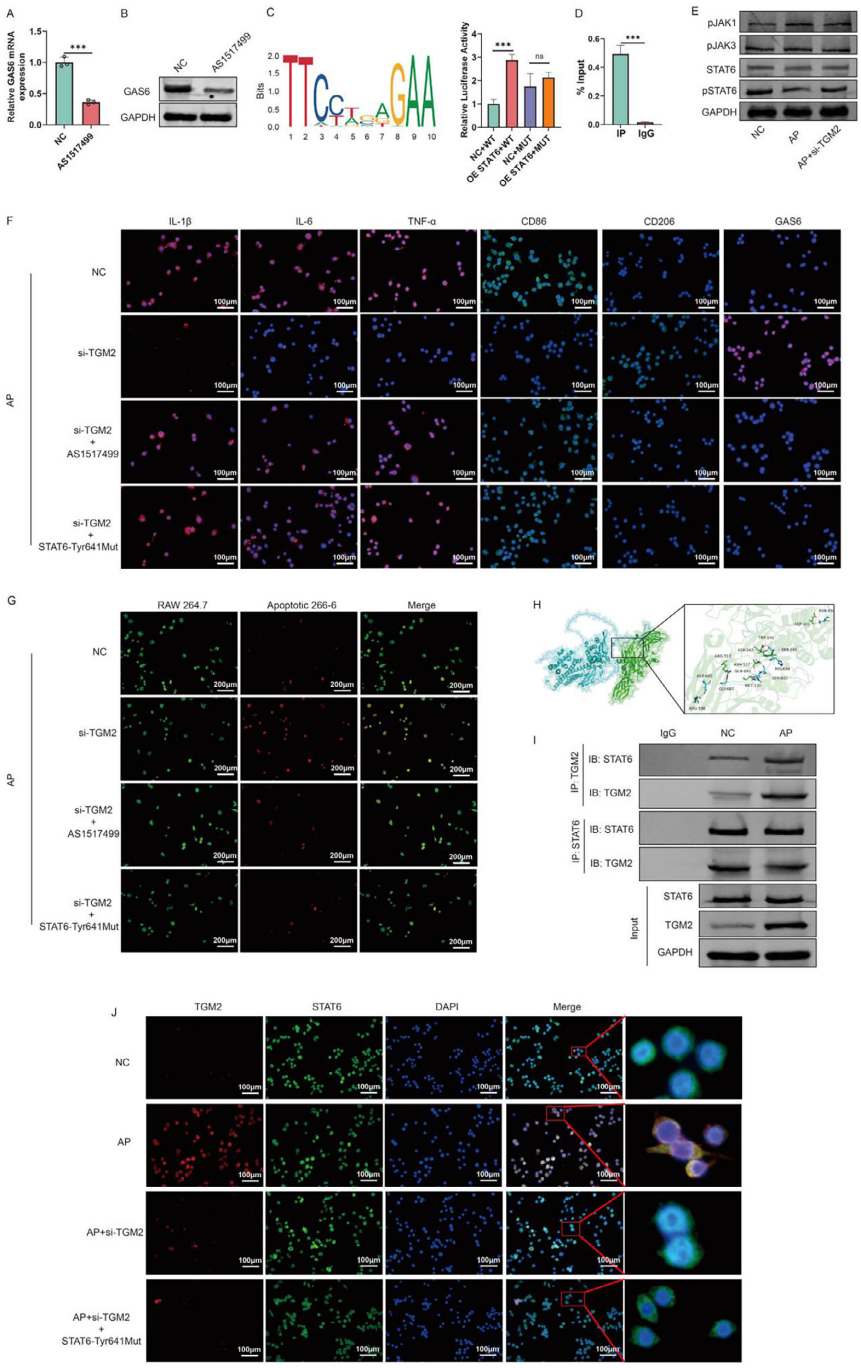
TGM2 downregulates GAS6 transcription by inhibiting STAT6 phosphorylation. (A,B) qPCR (A) and Western blot (B) analyses of GAS6 expression in RAW264.7 macrophages treated with STAT6 inhibitor (AS1517499) or left untreated (n = 3). (C) Dual‐luciferase reporter assay demonstrated that STAT6 regulates the transcription of GAS6 (n = 3). (D) ChIP‐qPCR confirmed that STAT6 regulates the transcription of GAS6 (n = 3). (E) Western blot results showing that TGM2 knockdown significantly restored the reduced pSTAT6 levels in macrophages stimulated with AP 266‐6 cell supernatants (n = 3). (F) IF analysis showing that STAT6 Tyr641 site phosphorylation inhibition abolished the suppressive effects of TGM2 knockdown on IL‐1β, IL‐6, TNF‐α, CD86, and CD206 expression in macrophages stimulated with AP 266‐6 supernatants (n = 3). (G) STAT6 Tyr641 site phosphorylation inhibition impaired the restoration of efferocytosis induced by TGM2 knockdown in macrophages treated with AP 266‐6 supernatants (n = 3). (H) Molecular docking model predicting the potential binding interface between TGM2 and STAT6. (I) Co‐immunoprecipitation (Co‐IP) analysis showing the interaction between TGM2 and STAT6 in RAW264.7 cells (IgG used as negative control) and the enhanced interaction upon AP supernatant stimulation (n = 3). (J) IF imaging results showed that TGM2 knockdown significantly restored the STAT6 nuclear translocation suppressed by stimulation with the supernatant of 266‐6 cells from the AP group, and this effect was abolished following the Tyr641 site mutation of STAT6 (n = 3). ^*^ *p* < 0.05, ^**^ *p* < 0.01, ^***^ *p* < 0.001.

**FIGURE 7:**
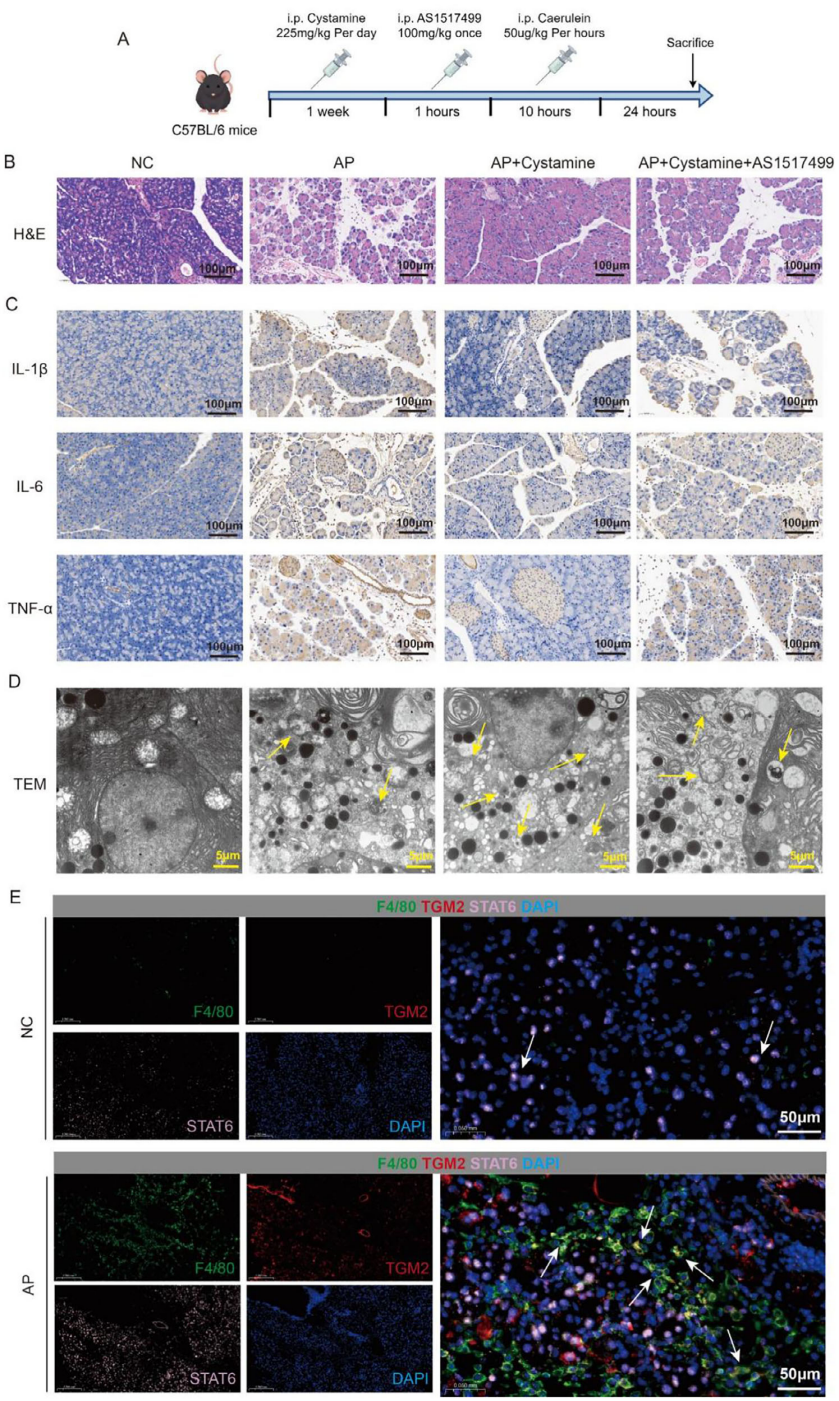
TGM2 promotes the aggravation of AP by inhibiting STAT6 phosphorylation. (A) Schematic diagram of the procedure for simultaneous inhibition of TGM2 and STAT6 in AP. (B,C) H&E (B) and IHC (C) staining showing that STAT6 inhibition counteracted the protective effects of Cystamine pretreatment on pancreatic injury and the reduction of IL‐1β, IL‐6, and TNF‐α expression in AP mice (n = 6). (D) TEM images showing that STAT6 inhibition weakened the Cystamine‐induced enhancement of efferocytosis in pancreatic tissues of AP mice (n = 3). (E) Multiplex immunofluorescence staining was performed to visualize the expression patterns of F4/80, TGM2, and STAT6 in the pancreatic tissues of mice from the NC and AP groups (n = 3).

Previous studies have demonstrated that STAT6 phosphorylation and nuclear translocation are key regulatory events that promote GAS6 transcription, thereby alleviating macrophage‐mediated inflammation [21]. Our experimental data confirmed that inhibition of STAT6 phosphorylation in macrophages resulted in a significant reduction in GAS6 expression at both the mRNA and protein levels (Figure 6A,B). In addition, STAT6 inhibition also reduced the mRNA levels of its downstream transcription factors Arg‐1, IL‐13, and CCL17 (Figure S9). A dual‐luciferase reporter assay demonstrated that STAT6 directly binds to the GAS6 gene promoter and regulates its transcriptional activity (Figure 6C)—an observation further validated via ChIP‐qPCR (Figure 6D). After stimulating macrophages with the culture supernatant of 266‐6 cells from the AP group, the expression level of total STAT6 protein in the cells remained unchanged, whereas the abundance of pSTAT6 (Tyr641) decreased significantly (Figure 6E). Notably, TGM2 knockdown reversed this effect and restored the expression level of pSTAT6 (Tyr641), and this effect was independent of pJAK1 and pJAK3, the upstream molecules of STAT6 (Figure 6E). To delineate the role of STAT6 Tyr641 phosphorylation in TGM2‐mediated regulation of macrophage function, we performed concurrent TGM2 knockdown and STAT6 inhibition or Tyr641 site mutation in macrophages prior to stimulation with AP‐conditioned medium. The effect of the Tyr641 site mutation of STAT6 is presented in Figure S10. The results showed that STAT6 inhibition and Tyr641 site mutation reversed the TGM2 knockdown‐induced downregulation of IL‐1β, IL‐6, TNF‐α, and CD86 expression, as well as the upregulation of CD206 expression (Figure 6F), and abolished the enhancement of efferocytic capacity elicited by TGM2 knockdown (Figure 6G). Molecular docking simulations predicted potential binding interfaces between TGM2 and STAT6 (Figure 6H). Co‐IP assays verified the existence of a physical interaction between these two proteins—an interaction that was markedly enhanced in the context of AP (Figure 6I). IF staining results showed that, compared with the control group, AP‐conditioned medium promoted the binding of TGM2 to STAT6 and inhibited the nuclear translocation of STAT6, whereas TGM2 knockdown restored the nuclear localization of STAT6 (Figure 6J). Notably, the Tyr641 site mutation of STAT6 reversed the aforementioned phenomena (Figure 6J). Concurrent inhibition of TGM2 and STAT6 in the mice model revealed that STAT6 inhibition similarly abrogated the protective effects of TGM2 inhibition against AP, including attenuation of pancreatic injury and downregulation of inflammatory cytokine expression (Figure 7A–C), while also diminishing the enhanced efferocytosis observed following TGM2 inhibition (Figure 7D). Multiplex immunofluorescence staining revealed that STAT6, which is expressed in macrophages, colocalized with TGM2 in the pancreatic tissues of AP models and did not translocate into the nucleus (Figure 7E). By contrast, in the NC group that lacked TGM2 and macrophages, STAT6 was expressed in the nucleus (Figure 7E). Collectively, these findings establish that TGM2 interacts with STAT6, thereby inhibiting its phosphorylation and nuclear translocation and the subsequent downregulation of GAS6 transcription—a mechanism that links TGM2 to STAT6‐dependent GAS6 transcription and efferocytosis impairment in experimental AP.

### 3.6 Construction of LF‐LNP@si‐TGM2 to Enhance Macrophage Efferocytosis and Alleviate Inflammation

**FIGURE 8:**
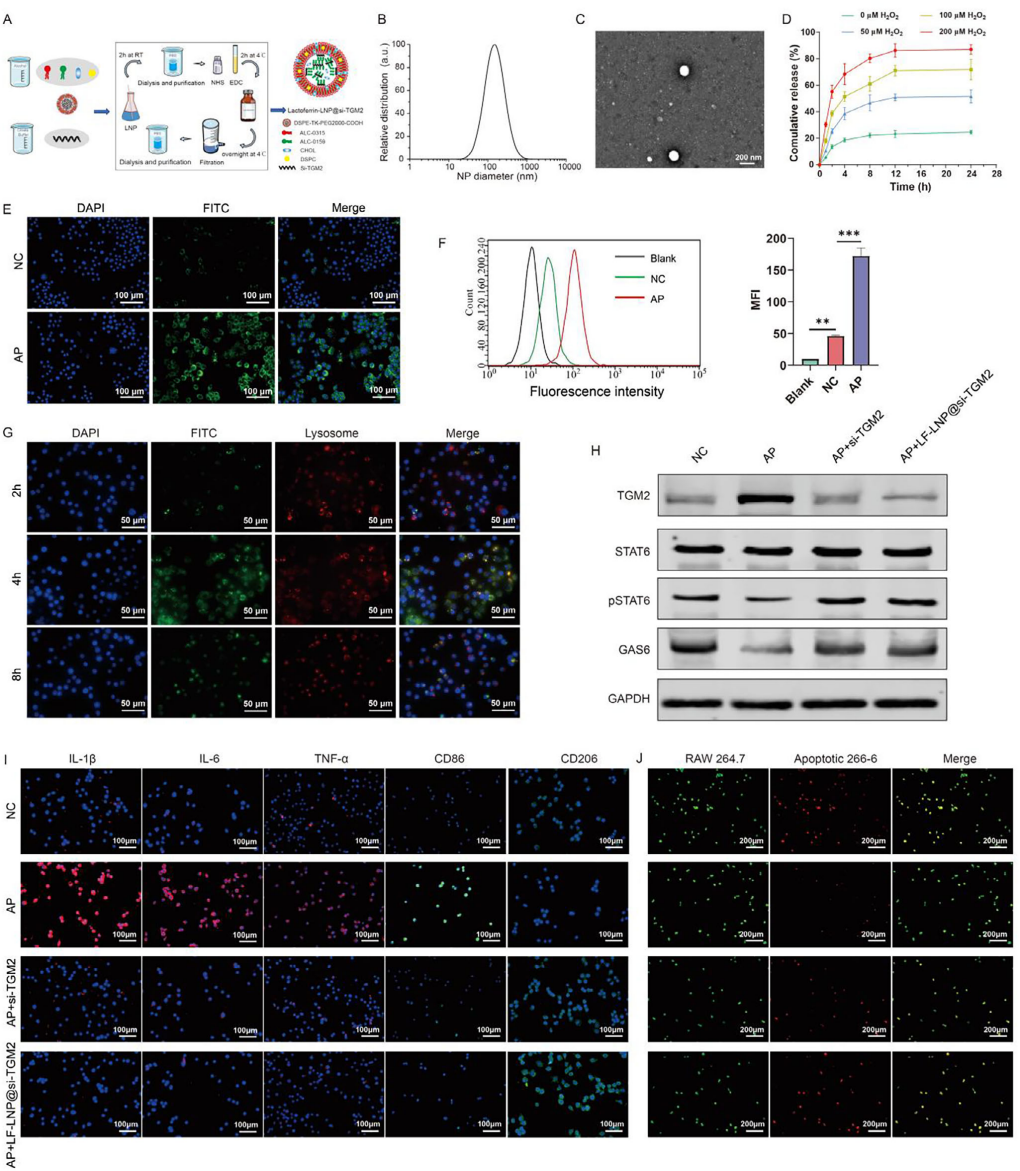
Preparation and in vitro functional validation of LF‐LNP@si‐TGM2. (A) Schematic illustration of the synthesis process of LF‐LNP@si‐TGM2. (B) Particle size distribution of LF‐LNP@si‐TGM2. (C) Transmission electron microscopy (TEM) image showing the morphology of LF‐LNP@si‐TGM2. (D) Time‐dependent FITC release curves of LF‐LNP@FITC under different H_2_O_2_ concentrations. (E,F) Fluorescence microscopy images (E) and flow cytometry analysis (F) showing RAW264.7 macrophages co‐incubated with LF‐LNP@FITC under stimulation with supernatants from NC or AP 266‐6 cells (n = 3). (G) Intracellular fluorescence tracking images showing the endocytosis and lysosomal escape process of LF‐LNP@si‐TGM2 in RAW264.7 macrophages (n = 3). (H) Western blot analysis showing the effects of LF‐LNP@si‐TGM2 and free si‐TGM2 on TGM2, pSTAT6, and GAS6 expression in macrophages (n = 3). (I) Immunofluorescence (IF) results demonstrating that both LF‐LNP@si‐TGM2 and si‐TGM2 reduced the upregulation of IL‐1β, IL‐6, TNF‐α, and CD86 induced by AP 266‐6 cell supernatants (n = 3). (J) LF‐LNP@si‐TGM2 and si‐TGM2 both restored the impaired efferocytosis of macrophages induced by AP 266‐6 cell supernatants (n = 3).

LF‐LNP@si‐TGM2 nanoparticles were prepared using a microfluidic mixing technique (Figure 8A). Dynamic light scattering analysis using the BI‐200 SM system (Brookhaven Instruments) revealed that the average particle size of LF‐LNP@si‐TGM2 was 156.36 ± 37.43 nm, with a ζ potential of 8.12 ± 2.06 mV (Figure 8B; Table 1). TEM showed that the nanoparticles exhibited a spherical morphology (Figure 8C). Furthermore, UV–vis spectrophotometric analysis determined an encapsulation efficiency of 95.27% (Table 1), confirming the successful loading of si‐TGM2 into the LNPs. To verify the ROS‐responsive release properties of LF‐LNP@si‐TGM2, nanoparticles loaded with the fluorescent probe FITC were incubated with different concentrations of H_2_O_2_ solutions, and FITC release was measured at 0, 1, 2, 4, 8, 12, and 24 h. The results demonstrated that the FITC release rate increased proportionally with H_2_O_2_ concentration, and reached a near‐maximum level at 12 h under each condition (Figure 8D). To further evaluate macrophage uptake and intracellular release behavior, FITC‐loaded LF‐LNP@si‐TGM2 was co‐incubated for 24 h with macrophages pretreated with conditioned media from either the NC or AP group 266‐6 cells. Both fluorescence microscopy and flow cytometry results demonstrated that the AP group exhibited significantly higher FITC release compared to the NC group (Figure 8E,F). Intracellular tracking experiments revealed that LF‐LNP@si‐TGM2 nanoparticles were internalized by macrophages within 2 h, localized to lysosomes at 4 h, and escaped from lysosomes by 8 h, indicating that the nanoparticles could avoid lysosomal degradation (Figure 8G). Taken together, these findings confirm that LF‐LNP@si‐TGM2 possesses ROS‐responsive characteristics and can be efficiently taken up by macrophages under AP conditions.

**TABLE 1:** The physicochemical characterization of Lactoferrin‐Modified siTGM2‐Loaded Lipid Nanoparticles.

Size(nm)PDI	156.36 ± 37.43 0.28
ζ potential(mV)	8.12 ± 2.06

Western blot results showed that LF‐LNP@si‐TGM2 significantly reduced the upregulated TGM2 expression induced by AP‐conditioned 266‐6 supernatant, with an even stronger effect than free si‐TGM2 (Figure 8H). Moreover, LF‐LNP@si‐TGM2 restored the expression of pSTAT6 and GAS6 in macrophages under AP conditions (Figure 8H). IF analysis further demonstrated that both LF‐LNP@si‐TGM2 and free si‐TGM2 reduced the expression of IL‐1β, IL‐6, TNF‐α, and CD86 in macrophages exposed to the AP‐conditioned environment (Figure 8I), while enhancing their efferocytic capacity (Figure 8J). Notably, the LF‐LNP@si‐TGM2 group exhibited superior efficacy compared with free si‐TGM2. Collectively, these findings indicate that LF‐LNP@si‐TGM2 efficiently targets macrophages under AP conditions, and by silencing TGM2, it restores the pSTAT6/GAS6 signaling axis, thereby enhancing macrophage efferocytosis and attenuating the inflammatory response. This strategy provides a promising therapeutic approach for the targeted treatment of AP.

### 3.7 LF‐LNP@si‐TGM2 Enhances Efferocytosis and Attenuates Disease Progression in AP Mice

**FIGURE 9:**
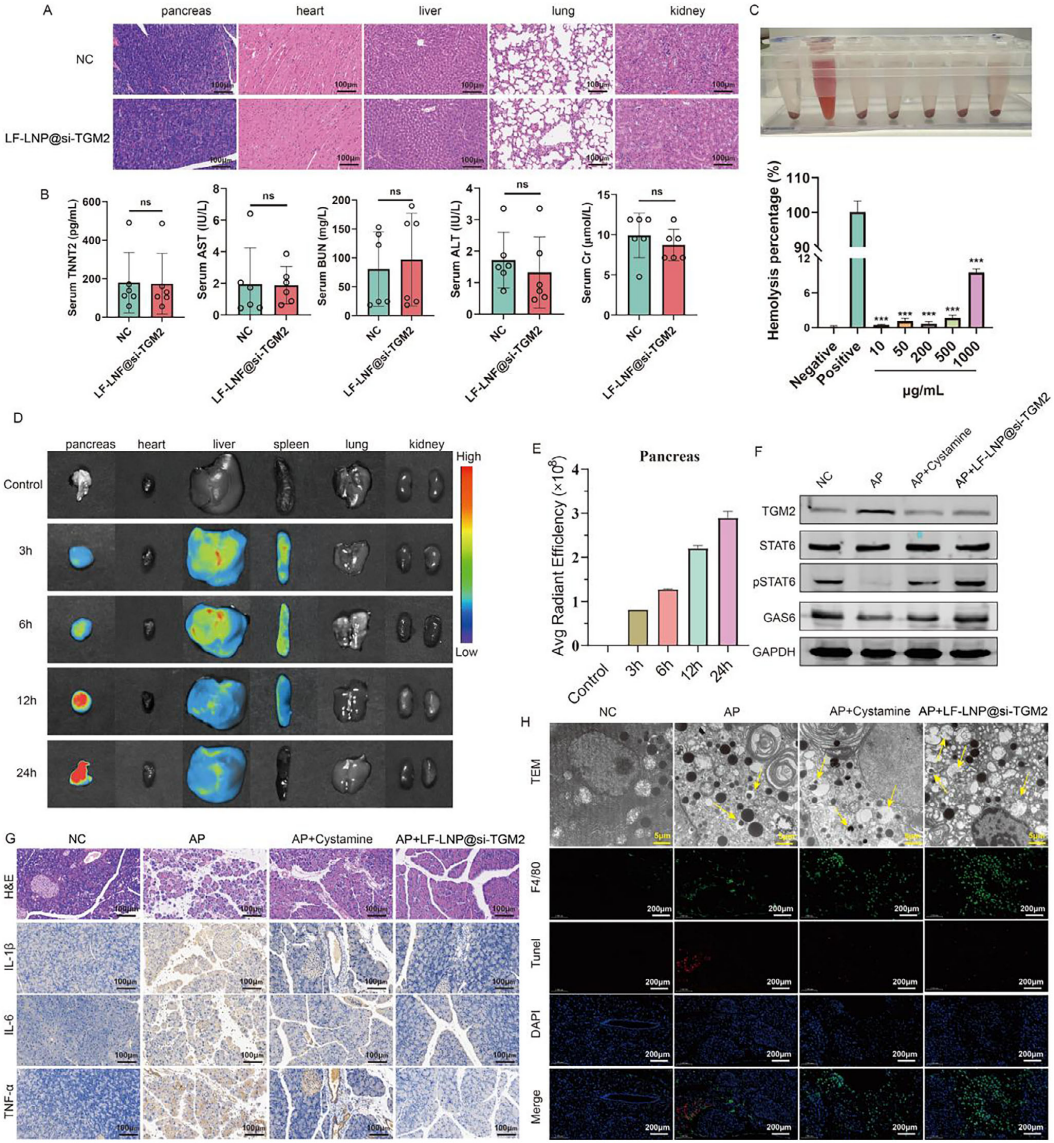
In vivo functional validation of LF‐LNP@si‐TGM2 in the AP mice model. (A) LF‐LNP@si‐TGM2 treatment did not cause damage to the pancreas, heart, liver, kidney, and lung of mice (n = 3). (B) LF‐LNP@si‐TGM2 treatment did not alter the serum levels of myocardial enzymes, alanine transaminase (ALT), aspartate transaminase (AST), creatinine (Cr), and blood urea nitrogen (BUN). (C) In vitro hemolysis assay of LF‐LNP@si‐TGM2 (n = 6). (D) In vivo imaging showing the organ distribution of LF‐LNP@si‐TGM2 at different time points after intravenous injection in AP mice (n = 3). (E) Distribution of LF‐LNP@si‐TGM2 in pancreatic tissues at various time points (n = 3). (F) Western blot analysis showing the effects of LF‐LNP@si‐TGM2 and Cystamine on TGM2, pSTAT6, and GAS6 expression in the pancreatic tissues of AP mice (n = 3). (G) H&E staining (left) and IHC (right) showing that both LF‐LNP@si‐TGM2 and Cystamine reduced pancreatic injury and decreased the expression levels of IL‐1β, IL‐6, and TNF‐α in pancreatic tissues of AP mice (n = 6). (H) Transmission electron microscopy (TEM) and IF results showing that both LF‐LNP@si‐TGM2 and Cystamine enhanced efferocytosis in the pancreatic tissues of AP mice (n = 3).

First, we evaluated the in vivo biosafety of LF‐LNP@si‐TGM2. LF‐LNP@si‐TGM2 did not cause damage to the pancreas, heart, liver, lung, and kidney of mice (Figure 9A), and the serum levels of myocardial enzymes, alanine transaminase (ALT), aspartate transaminase (AST), creatinine (Cr), and blood urea nitrogen (BUN) remained unchanged after LF‐LNP@si‐TGM2 treatment (Figure 9B). In vitro hemolysis assays showed that LF‐LNP@si‐TGM2 at concentrations of 10, 50, 200, 500, and 1000 µg/mL did not induce significant hemolytic reactions (Figure 9C). After establishing the AP mice model, LF‐LNP@si‐TGM2 was administered via tail vein injection. At 3, 6, 12, and 24 h post‐injection, major organs including the kidneys, lungs, spleen, liver, heart, and pancreas were harvested for in vivo imaging analysis (Figure 9D). The results demonstrated that LF‐LNP@si‐TGM2 predominantly accumulated in pancreatic tissue, with only minor distribution in the liver and spleen, where it was rapidly cleared. No significant accumulation was detected in other organs (Figure 9E), indicating that under AP conditions, LF‐LNP@si‐TGM2 effectively targets the inflamed pancreas and releases its payload efficiently. Western blot analysis revealed that both LF‐LNP@si‐TGM2 and cystamine reduced TGM2 expression in the pancreatic tissues of AP mice, while increasing the expression levels of pSTAT6 and GAS6 (Figure 9F). Further H&E staining and IHC analyses confirmed that both LF‐LNP@si‐TGM2 and Cystamine alleviated pancreatic tissue injury and reduced the expression of inflammatory cytokines in AP mice (Figure 9G). TEM and IF analyses revealed that both LF‐LNP@si‐TGM2 treatment and Cystamine treatment enhanced efferocytosis in pancreatic tissues of AP mice, with LF‐LNP@si‐TGM2 exhibiting a superior effect (Figure 9H). Collectively, these findings demonstrate that LF‐LNP@si‐TGM2 specifically targets the inflamed pancreatic tissue in AP mice and ameliorates pancreatic injury and systemic inflammation, in association with restored macrophage efferocytic readouts. The aforementioned therapeutic effects of LF‐LNP@si‐TGM2 were also verified in SAP mice, which were characterized by decreased TGM2 expression, STAT6 activation, elevated GAS6‐dependent efferocytosis levels, and reduced pancreatic and systemic inflammation levels. Collectively, these in vivo results demonstrate that LF‐LNP@si‐TGM2 achieves pancreatic accumulation and target engagement, with concomitant improvements in inflammatory and histopathological readouts in experimental AP and SAP models.

## 4 Discussion

The progression of AP toward severe disease is driven by a dynamic feed‐forward loop linking local pancreatic injury to systemic inflammation and subsequent immune dysregulation [5, 14]. Importantly, most patients with mild AP recover with standard supportive management, including timely fluid resuscitation, analgesia, nutritional support, and complication‐oriented care [5]. Thus, the major unmet clinical need lies in preventing early deterioration in patients at risk of developing SAP and in attenuating the inflammatory–immune derailment that accompanies pancreatic necrosis and persistent organ failure [14]. Once extensive necrosis and/or infected necrosis is established, outcomes are frequently shaped by complications that may require procedural or surgical management; therefore, mechanism‐based immunomodulatory strategies should be viewed as adjunctive interventions within defined therapeutic windows, rather than replacements for established supportive and interventional care [22]. In this study, integrating multi‐omics screening, mechanistic validation, and a targeted delivery approach, we identify TGM2 as a disease‐associated regulator that amplifies macrophage‐driven inflammation and perturbs pro‐resolving programs. We further develop a lactoferrin‐modified, ROS‐responsive lipid nanoparticle formulation (LF‐LNP@si‐TGM2) to enable macrophage‐addressable gene silencing in inflamed pancreatic tissue. Collectively, these data nominate TGM2 as a mechanistically relevant target and provide a proof‐of‐concept nanotherapeutic strategy that may complement current management, particularly in settings linked to severe progression.

TGM2 is a multifunctional enzyme that catalyzes the cross‐linking reaction between glutamine and lysine residues, thereby participating in various biological processes, including cell growth, differentiation, death, inflammation, tissue repair, and fibrosis [23]. During inflammation, the expression and activity of TGM2 are often upregulated, and it participates in the initiation, maintenance, and regulation of inflammatory responses through multiple mechanisms, making it a key molecule in various inflammation‐related diseases. In a psoriasis model, TGM2 activates the NF‐κB signaling pathway, leading to the increased expression of inflammatory mediators such as IL‐6, CCL20, and CXCL8. This upregulation promotes the recruitment of CCR6^+^ γδ T cells and neutrophils, thereby exacerbating skin inflammation [24]. Similarly, in ultraviolet‐induced skin inflammation, activation of TGM2 enhances the phosphorylation of p65, which in turn increases the transcriptional activity of NF‐κB, leading to the overexpression of pro‐inflammatory cytokines [25]. The regulatory role of TGM2 in inflammation also involves its interactions with other molecules and signaling pathways. For instance, in neuroinflammation, treatment with TGM2 inhibitors alleviates LPS‐induced neuroinflammatory responses by suppressing the AKT/mTOR signaling pathway, thereby reducing the production of inflammatory mediators and neuronal damage [26]. These findings indicate that TGM2 plays a multilayered regulatory role in inflammation, yet its specific function in AP has remained unclear. In this study, we demonstrated that TGM2 expression was significantly upregulated in a caerulein‐induced AP mice model, while treatment with its inhibitor Cystamine markedly attenuated pancreatic injury. These results provide direct evidence for the pro‐inflammatory role of TGM2 in the progression of AP. Taken together, our findings suggest that TGM2 is not only a potential biomarker for AP but also a key molecular regulator driving disease severity, thereby filling a critical gap in understanding the function of TGM2 in AP pathogenesis.

Macrophages act as central regulators within the inflammatory microenvironment of AP, and the imbalance between their pro‐inflammatory and anti‐inflammatory activities directly determines the direction and severity of the inflammatory response [27, 28]. Importantly, in severe AP, necrosis of acinar cells is a defining pathological feature and a major source of DAMP‐driven inflammatory amplification [7, 10]. Apoptosis is generally less inflammatory and may be associated with a more contained injury phenotype; however, when apoptotic cells are not efficiently cleared, they can undergo secondary necrosis, further increasing DAMP burden [7, 8, 10]. Thus, impaired efferocytosis is best interpreted as an amplifier of inflammatory persistence that hinders resolution, rather than as the primary initiating event in AP [10, 11]. In vitro experiments confirmed that stimulation of RAW264.7 macrophages with supernatants from AP‐induced acinar cells led to a marked upregulation of TGM2 expression, accompanied by increased levels of pro‐inflammatory cytokines and the M1 macrophage marker CD86. Conversely, silencing TGM2 significantly reversed these changes. These findings suggest that TGM2 promotes macrophage polarization toward a pro‐inflammatory phenotype, serving as a critical driver of inflammatory damage during the progression of AP [29, 30]. In addition to promoting macrophage polarization toward a pro‐inflammatory phenotype, we found that TGM2 also impairs the efferocytic function of macrophages. Efferocytosis — the process by which macrophages engulf and remove apoptotic cells — is a critical mechanism for limiting inflammatory spread. When this function is compromised, apoptotic cells accumulate and continuously release pro‐inflammatory signals, thereby creating a vicious cycle of “inflammation–efferocytosis defect” [28]. Macrophages recognize the “eat‐me” signals displayed on the surface of apoptotic cells — such as phosphatidylserine (PS) and calreticulin — through multiple receptors. These signals are transmitted via bridging molecules, including MFG‐E8 and Gas6, which bind to corresponding receptors on the macrophage surface, thereby initiating the efferocytosis process [10, 31]. However, when the expression or function of these receptors or bridging molecules is impaired, apoptotic cells cannot be efficiently cleared. As a result, these cells undergo secondary necrosis, releasing large amounts of pro‐inflammatory molecules, which in turn trigger or exacerbate inflammatory responses [32, 33]. As a bridging molecule in the efferocytosis process, GAS6 binds to its receptors Tyro3, Axl, and MerTK—members of the TAM receptor family—to regulate multiple steps of apoptotic cell clearance.

Specifically, GAS6 interacts with TAM receptors to facilitate macrophage recognition and engulfment of apoptotic cells. For example, in models of acute lung injury, administration of recombinant GAS6 (rGas6) has been shown to significantly enhance the ability of alveolar macrophages to clear apoptotic cells, thereby reducing inflammatory injury [34]. Second, the GAS6/TAM signaling pathway regulates the efficiency of efferocytosis and the inflammatory response by modulating downstream molecules such as the NLRP3 inflammasome. In sepsis models, overexpression of GAS6 has been shown to inhibit NLRP3 activation, thereby reducing cardiomyocyte apoptosis and mitochondrial damage, ultimately improving cardiac function and alleviating systemic inflammation [35]. This study found that in TGM2^+^ macrophages, the expression of the key efferocytosis molecule GAS6 was significantly downregulated, while knockdown of TGM2 restored both GAS6 levels and efferocytic capacity. More importantly, simultaneous knockdown of GAS6 completely abolished the beneficial effects of TGM2 silencing on efferocytosis and inflammation, indicating that GAS6 serves as the critical downstream effector of TGM2‐mediated regulation of efferocytosis. Consistent with these findings, Zhu et al. reported that in AP, CD47 expressed on the surface of pancreatic acinar cells acts as an “eat‐me” signal, promoting macrophage‐mediated efferocytosis and thereby exerting a self‐protective effect during inflammation [36]. Moreover, its upstream TRIM28/miR‐133a regulatory axis can suppress CD47 expression, thereby impairing the efferocytosis process and consequently exacerbating the severity of AP [36]. Our study proposes an independent regulatory axis distinct from their findings, in which TGM2 impairs macrophage efferocytosis by downregulating GAS6, a key bridging molecule essential for the efferocytosis process. Through this mechanism, TGM2 indirectly inhibits the clearance of apoptotic cells, thereby sustaining inflammation and aggravating the progression of AP.

Previous studies have demonstrated that pSTAT6 can translocate into the nucleus and bind to the GAS6 promoter, directly driving its transcription and thereby promoting macrophage efferocytosis. In models of acute lung injury (ALI), activation of STAT6 phosphorylation induces alveolar macrophages (AMΦs) to express GAS6 through downstream signaling pathways, consequently enhancing their ability to engulf apoptotic neutrophils [21]. Moreover, pSTAT6‐mediated GAS6 expression has been shown to play a crucial role in inflammation resolution after brain injury. Studies have found that the EphA4 receptor suppresses the ERK/STAT6 signaling pathway, thereby reducing the expression of MERTK and GAS6, which in turn impairs the ability of monocytes/macrophages to clear apoptotic debris [37]. However, the regulatory mechanism of the STAT6–GAS6 axis in AP remains unclear. In this study, through a series of molecular experiments, we demonstrated for the first time that TGM2 directly interacts with STAT6, thereby inhibiting its phosphorylation and nuclear translocation, which in turn downregulates GAS6 transcription. This discovery not only fills the molecular gap in understanding the regulation of efferocytosis in AP but also establishes a novel pathological signaling pathway — the “TGM2–STAT6–GAS6” axis, providing key mechanistic insights into the progression of SAP. One limitation of our study is that we did not further explore how TGM2 suppresses STAT6 phosphorylation. Notably, Chen Xiaoping et al. reported that TGM2, as a transglutaminase, can mediate histone serotonylation, thereby promoting MYC transcription and exacerbating hepatocarcinogenesis [38]. However, whether the inhibitory effect of TGM2 on STAT6 is associated with this emerging form of post‐translational modification remains to be further investigated.

RNA interference (RNAi) technology provides a tool for targeted therapy of AP by specifically silencing target genes; however, naked siRNA suffers from poor stability and insufficient targeting capability [39, 40]. The LF‐LNP@si‐TGM2 delivery system constructed in this study achieves precise intervention in AP through a multifaceted design.

First, LF‐LNP@si‐TGM2 exhibits a particle size of 156.36 ± 37.43 nm, a ζ‐potential of 8.12 ± 2.06 mV, and an encapsulation efficiency of 95.27%, meeting the physicochemical criteria of an ideal drug delivery system [41, 42]. Second, lactoferrin can specifically bind to lactoferrin receptors that are highly expressed on the surface of macrophages [43], thereby markedly enhancing the targeting capability of the carrier toward inflammatory macrophages. Notably, Fang et al. constructed macrophage membrane‐camouflaged lipid nanoparticles, with mRNA of Annexin A1 encapsulated therein to activate the macrophage efferocytosis process and alleviate AP [44]. In contrast to the approach employed by Fang et al., which leverages surface adhesion molecules of macrophage membranes to home liposomes to inflammatory sites, our strategy achieves macrophage targeting through lactoferrin modification. Future studies may integrate multiple targeting strategies to further achieve multiplexed targeting. Subsequently, its ROS‐responsive property ensures the precise release of si‐TGM2 at inflammatory sites, while normal cells are protected from the unpredictable side effects associated with TGM2 inhibition. Finally, in terms of functionality, LF‐LNP@si‐TGM2 not only silences TGM2 more efficiently (with superior effects compared to free si‐TGM2), but also restores the pSTAT6/GAS6 signaling axis, significantly enhancing macrophage efferocytosis and reducing inflammatory cytokine levels. Its therapeutic efficacy surpasses that of the traditional TGM2 inhibitor Cystamine. In conclusion, LF‐LNP@si‐TGM2, through its dual design of ROS‐responsive release and lactoferrin‐mediated targeting, achieves precise intervention of pancreatic macrophages in AP, providing a translationally applicable strategy for targeted therapy of the disease.

This study also has several limitations. First, in the MR analysis section, since the public database used did not subdivide the etiologies of AP, we were unable to exclude the potential impacts of different etiologies on TGM2 expression. Given that AP induced by distinct etiologies may exhibit heterogeneity in the early inflammatory microenvironment and stress response, the regulatory effect of TGM2 in the context of specific etiologies warrants further verification through stratified studies in future research. Second, we did not explore the binding regions and sites between TGM2 and STAT6, which requires more detailed investigations in subsequent studies.

## 5 Conclusion

This study, for the first time, demonstrates that TGM2 interacts with STAT6 to inhibit its phosphorylation, thereby downregulating GAS6 transcription, which leads to impaired macrophage efferocytosis and ultimately exacerbates the severity of AP. Based on this mechanism, we developed a ROS‐responsive lactoferrin‐modified lipid nanoparticle system (LF‐LNP@si‐TGM2) that can specifically target pancreatic macrophages in AP. By silencing TGM2, this system restores the STAT6–GAS6 signaling axis, effectively alleviating pancreatic inflammation and tissue injury. These findings provide mechanistic insight into macrophage‐linked inflammatory persistence in experimental pancreatitis and support TGM2 as a candidate target for stratified, mechanism‐informed adjunctive therapy—intended to complement, rather than replace, standard supportive management and the procedural/surgical care required for necrotizing or infected complications.

## Funding

This study was supported by the National Natural Science Foundation of China (No. 82370650) and the Key Research and Development Program of Heilongjiang Province (No. 2022ZX06C06).

## Ethics Statement

The study was approved by the ethics committee of the First Affiliated Hospital of Harbin Medical University (No. YS447).

## Conflicts of Interest

The authors declare no conflicts of interest.

## Supporting information




**Supporting File 1**: advs74483‐sup‐0001‐FigureS1‐S11.pdf.

**Supporting File 2**: advs74483‐sup‐0002‐TableS1‐S3.docx.

## Data Availability

The data that support the findings of this study are openly available in [NAME] at [URL/DOI], reference number [REF]. These data were derived from the following resources available in the public domain: [Resource 1], https://www.[resource1]; [Resource 2], https://www.[resource2].
