# Supplementary material for: TGM2 Aggravates Acute Pancreatitis by Impairing Macrophage Efferocytosis Through Inhibition of the STAT6–GAS6 Axis
Source: Adv Sci (Weinh). 2026 Feb 17;13(24):e20739. doi: 10.1002/advs.202520739 (PMC13116110; doi:10.1002/advs.202520739)

Supplementary Figure 1: Correlation between TGM2 expression and infiltration scores of various immune cell types from immune infiltration analysis in the mouse Bulk RNA-seq dataset.

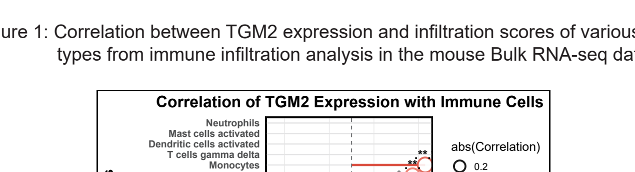

Supplementary Figure 2: TGM2 is upregulated in severe acute pancreatitis (SAP)

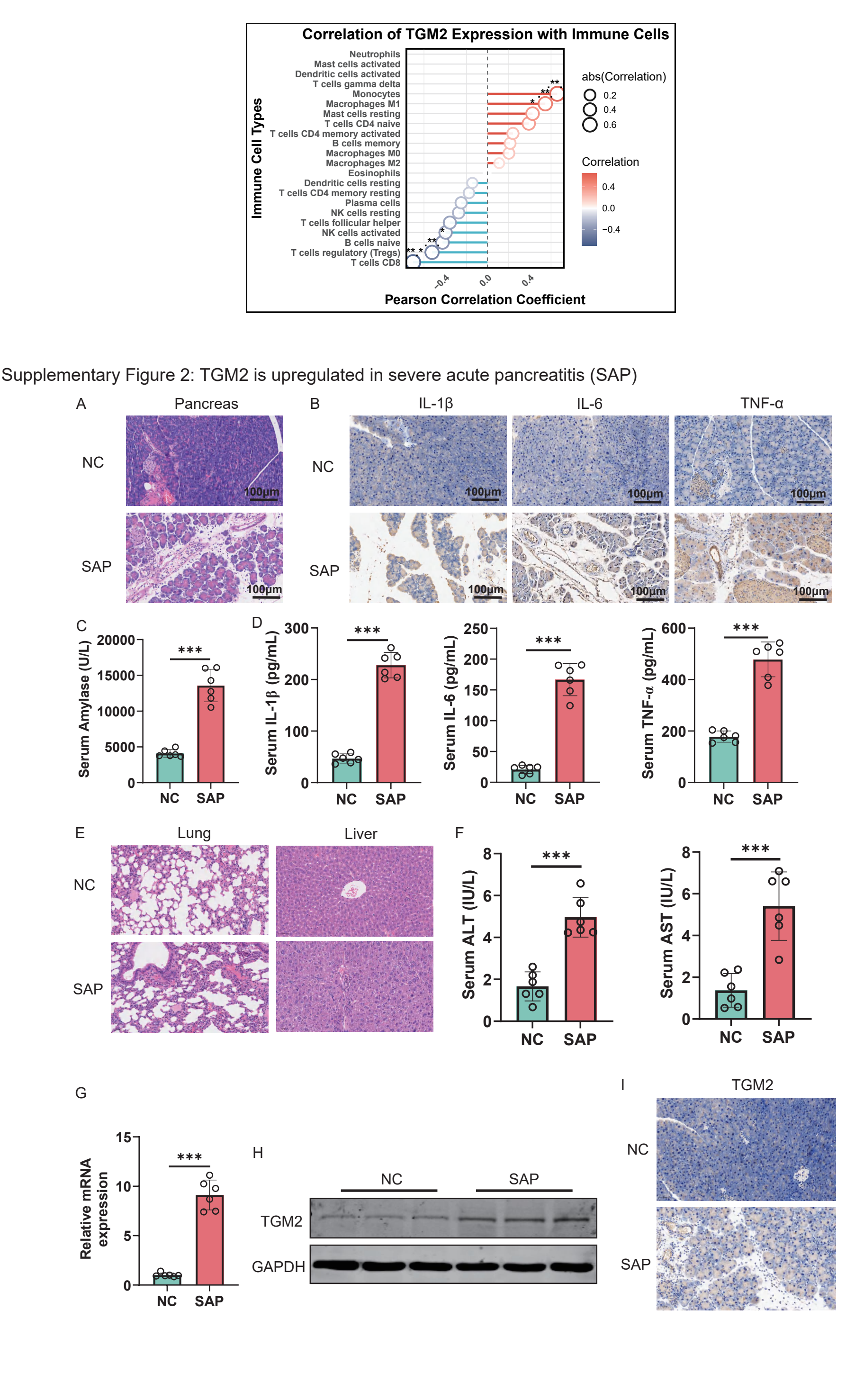

Supplementary Figure 3: TGM2 inhibition attenuates local and systemic inflammatory levels in SAP

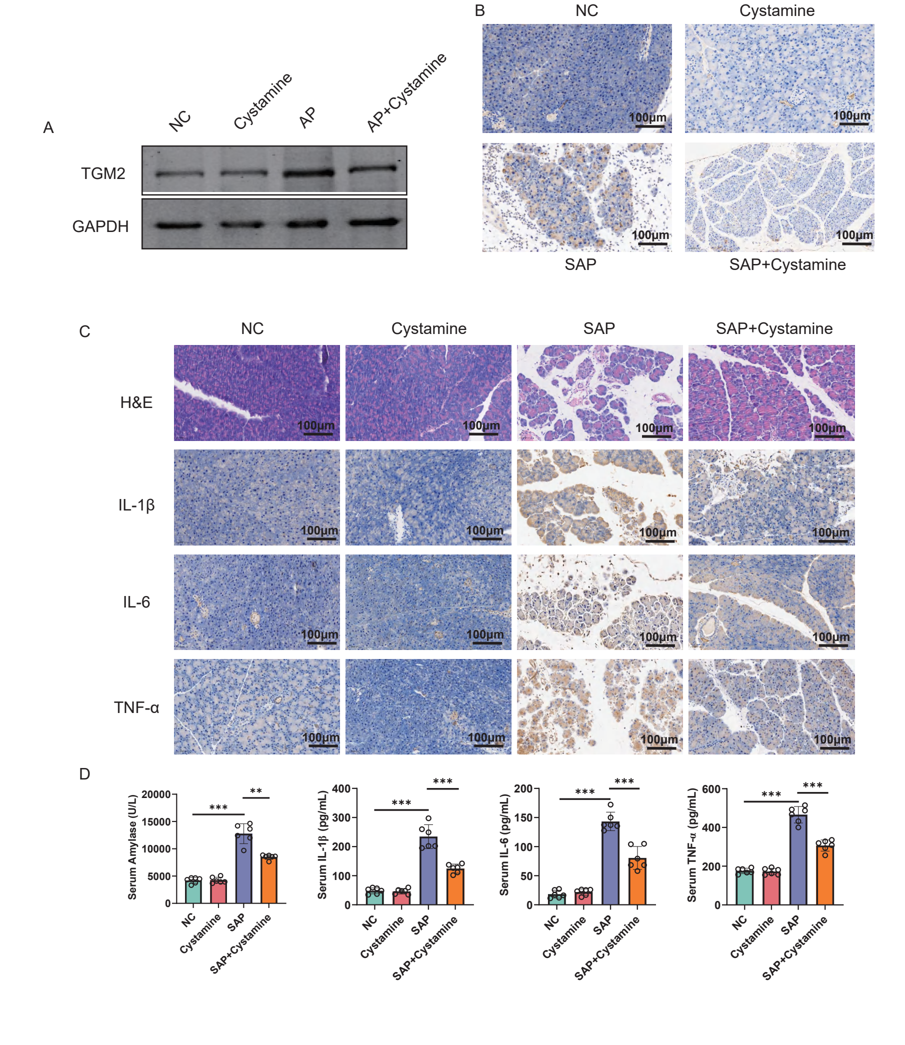

Supplementary Figure 4: Analysis of mouse single-cell RNA-seq data.

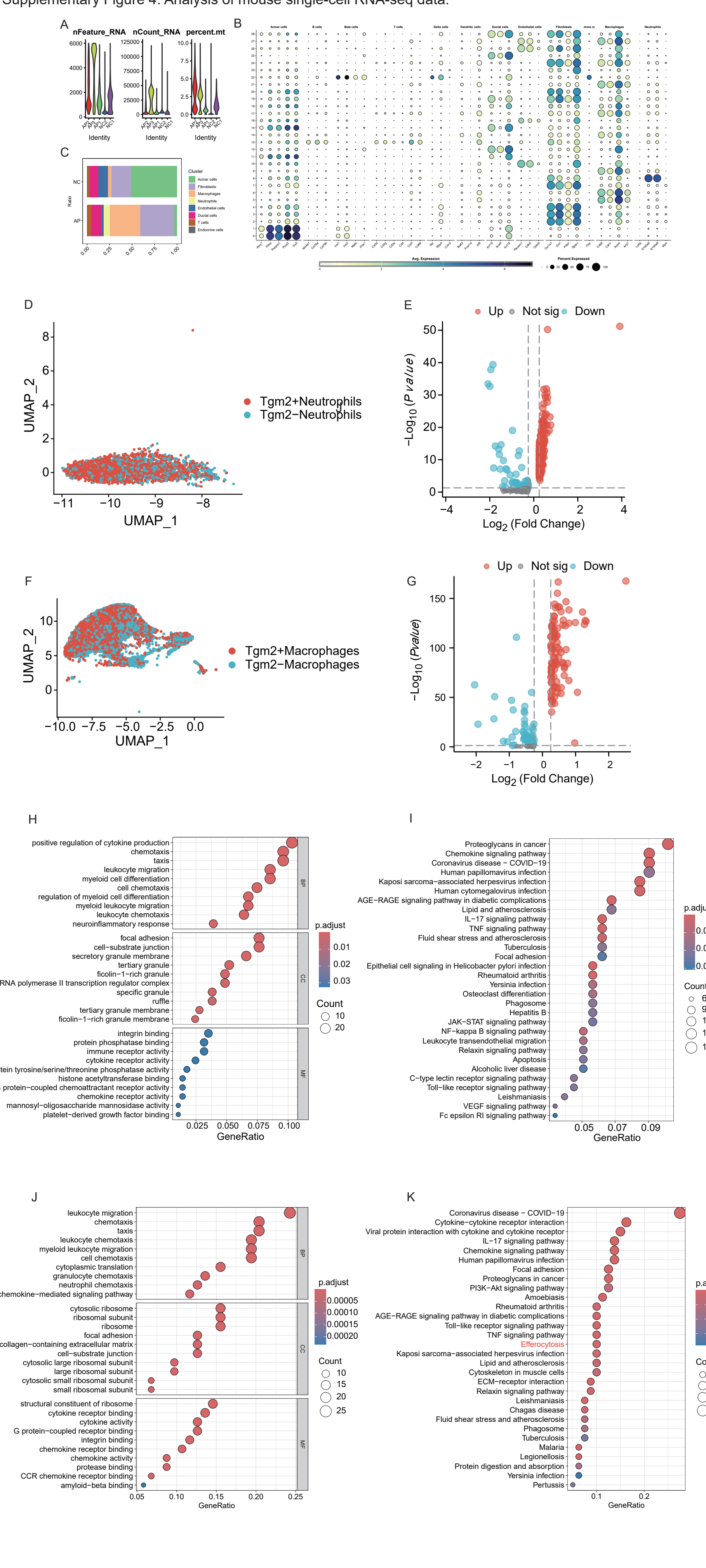

Supplementary Figure 5: Analysis of cell-cell communication between TGM2+ neutrophils/TGM2+ neutrophils and other cells from mouse single-cell RNA-seq data.

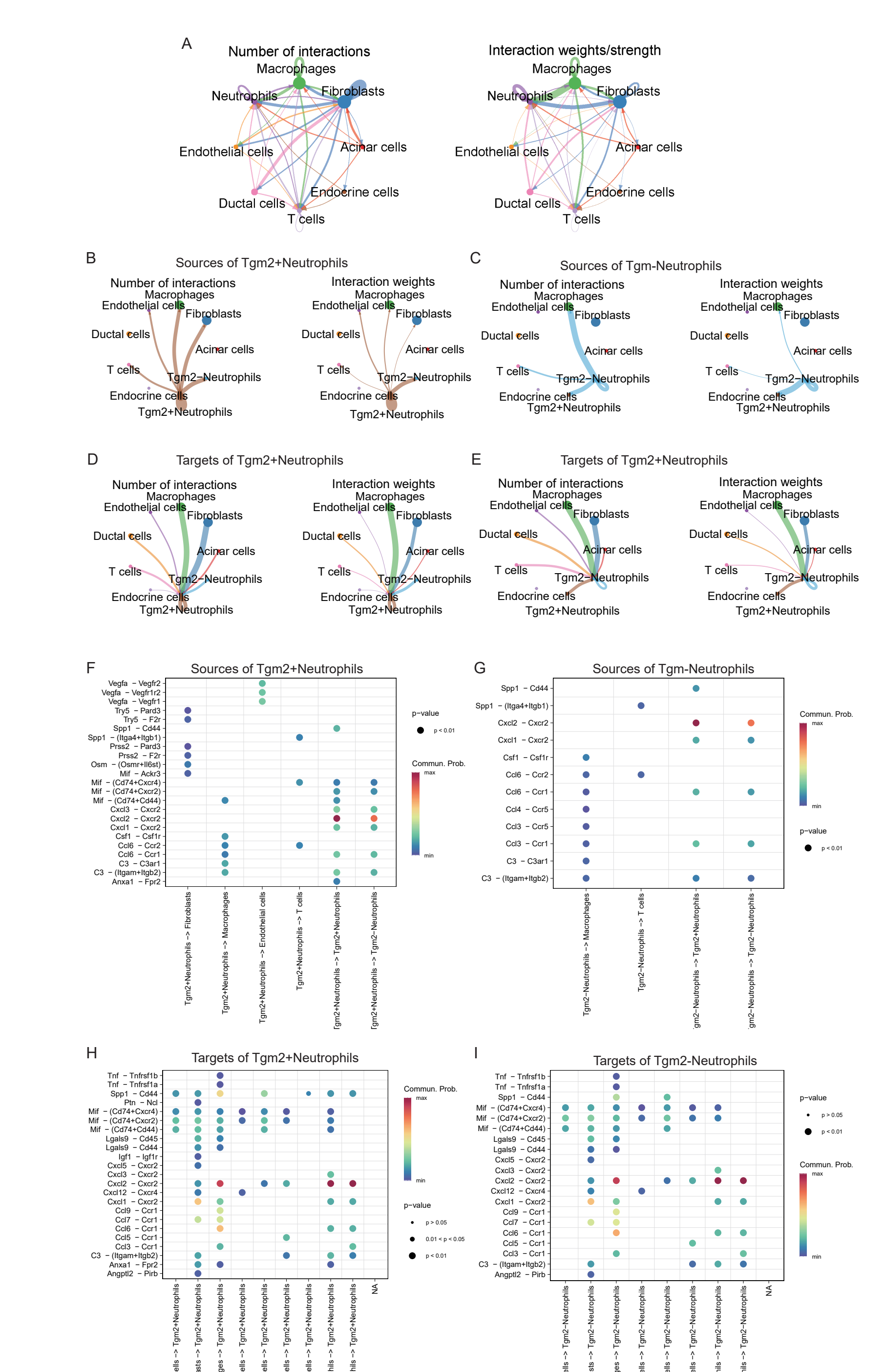

Supplementary Figure 6: Analysis of cell-cell communication between TGM2+ macrophages/TGM2+ macrophages and other cells from mouse single-cell RNA-seq data.

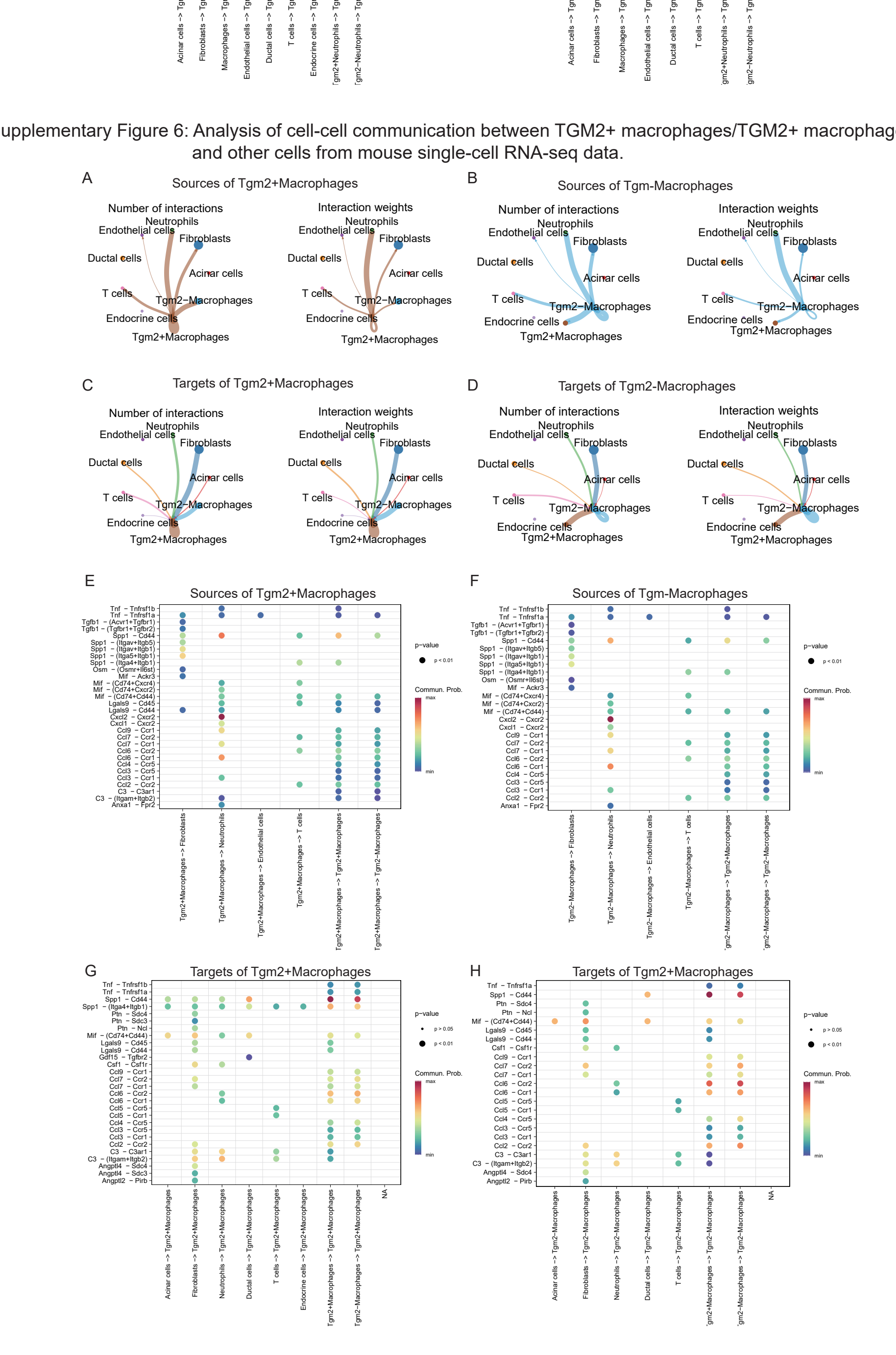

Supplementary Figure 7: TGM2 exacerbates the inflammatory level of macrophages in the context of AP in a GAS6-dependent manner.

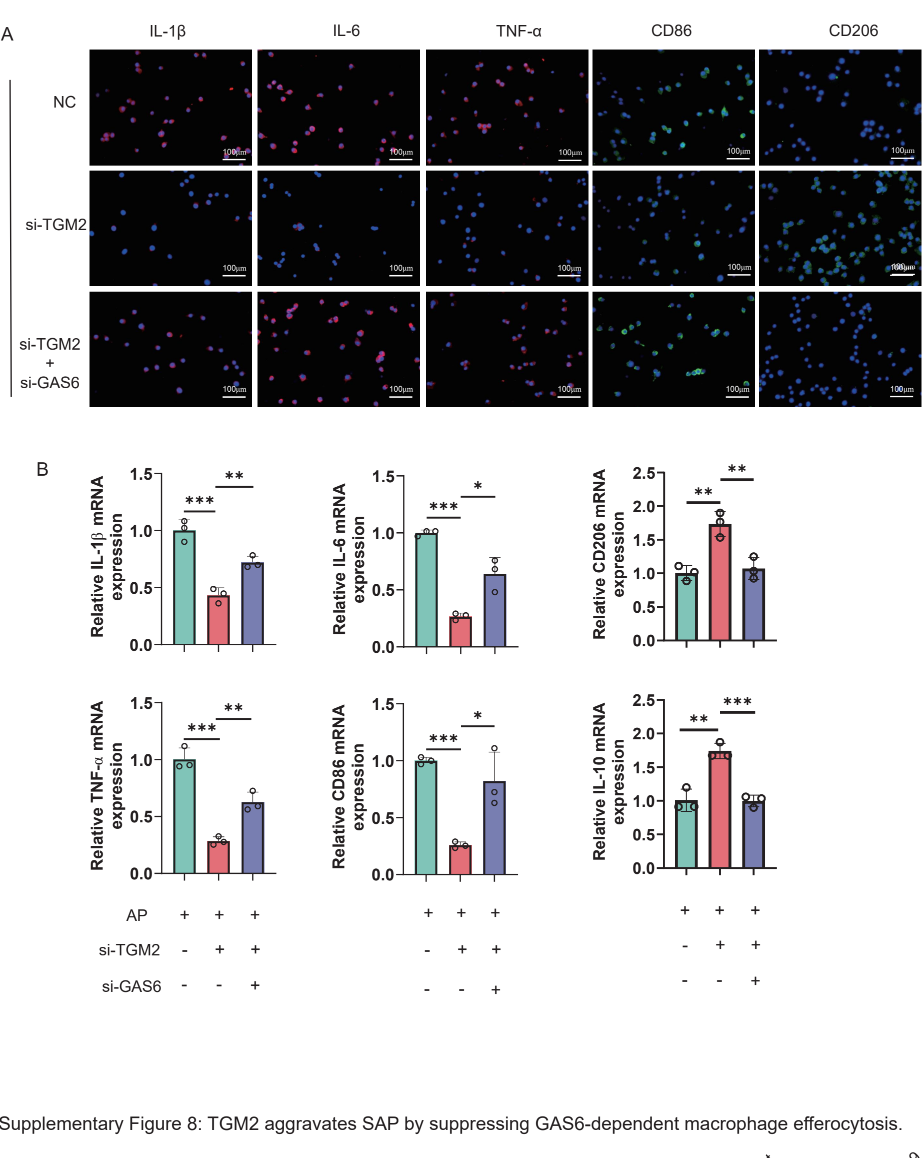

Supplementary Figure 8: TGM2 aggravates SAP by suppressing GAS6-dependent macrophage efferocytosis.

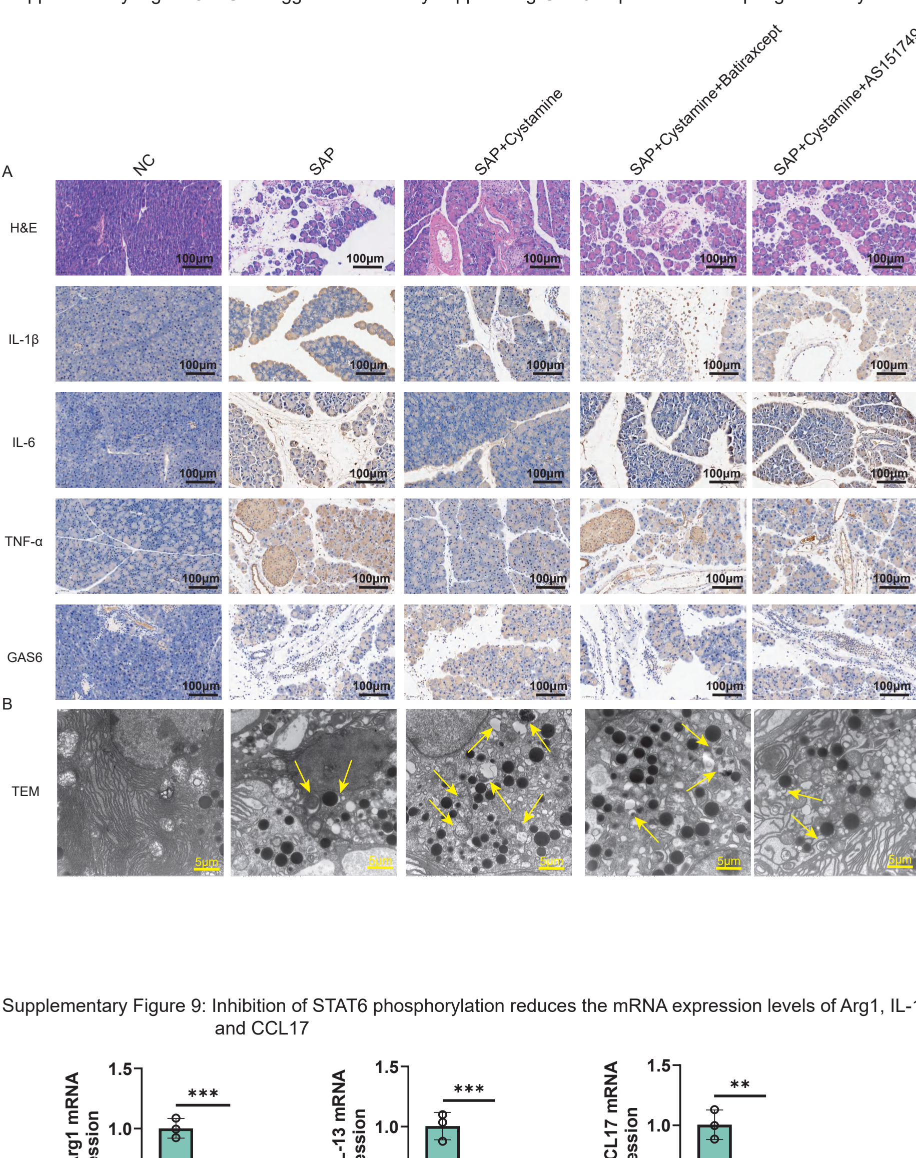

Supplementary Figure 9: Inhibition of STAT6 phosphorylation reduces the mRNA expression levels of Arg1, IL-13 and CCL17

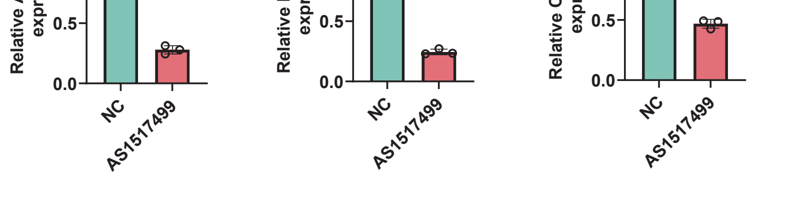

Supplementary Figure 10: Detection of STAT6-Tyr641 site mutation efficiency in Raw264.7 cell line

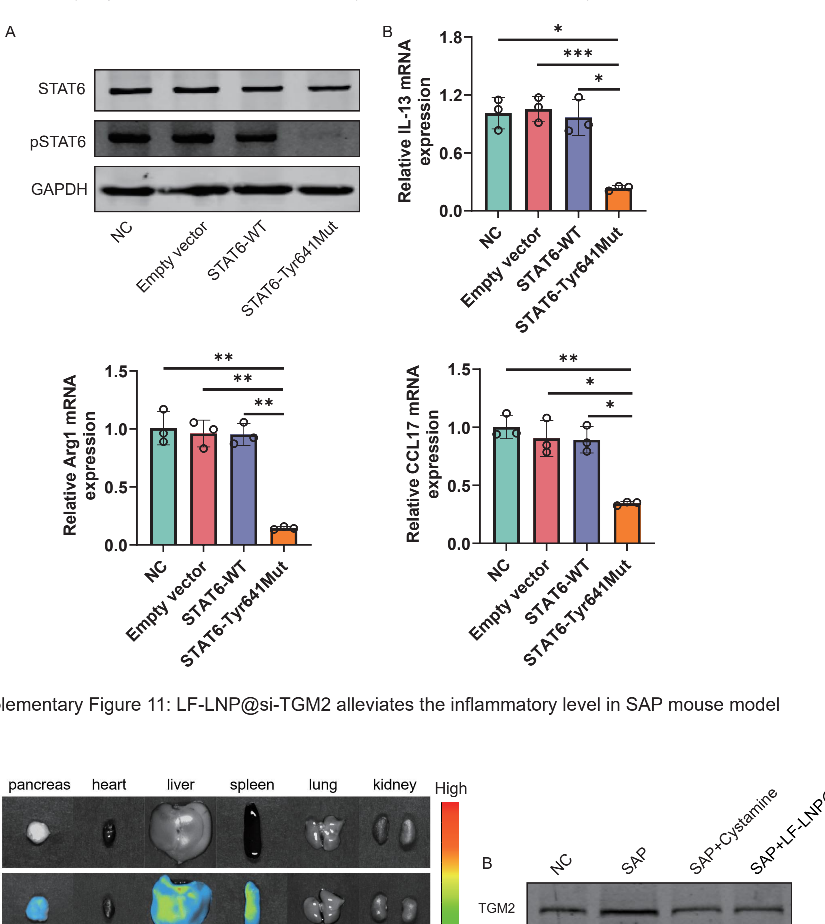

Supplementary Figure 11: LF-LNP@si-TGM2 alleviates the inflammatory level in SAP mouse model

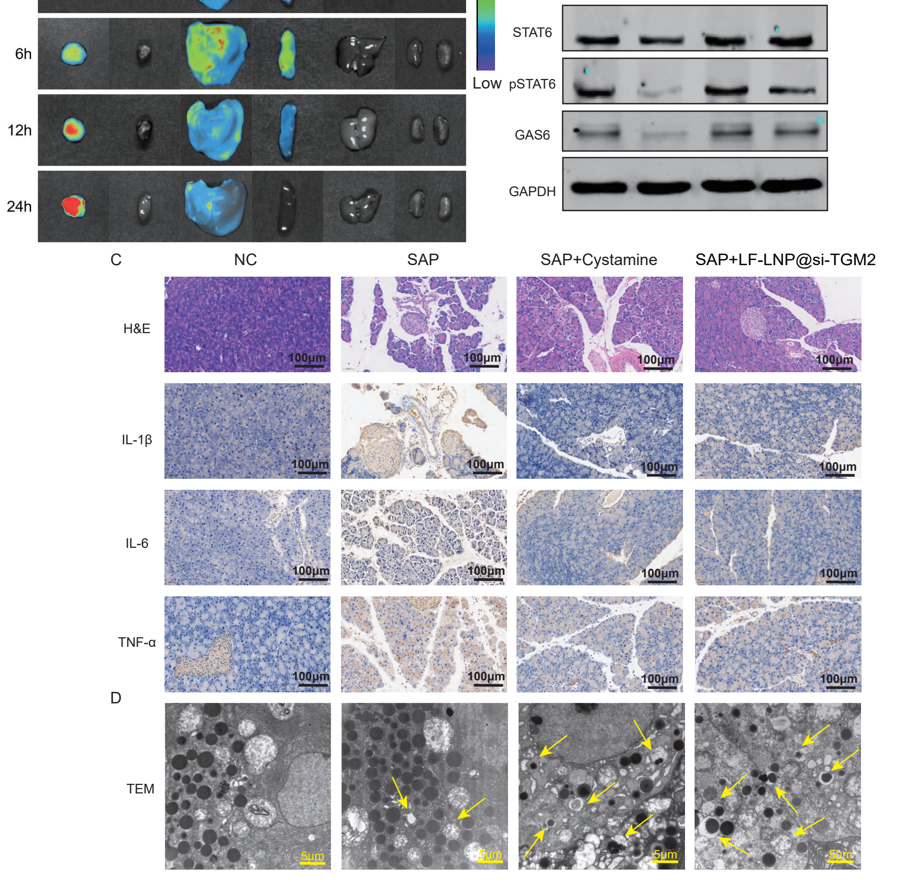

Supplement: Supplementary file 1 — Supporting File 1: advs74483‐sup‐0001‐FigureS1‐S11.pdf. [file ADVS-13-e20739-s001.pdf]
