# Supplementary material for: TGM2 Aggravates Acute Pancreatitis by Impairing Macrophage Efferocytosis Through Inhibition of the STAT6–GAS6 Axis
Source: Adv Sci (Weinh). 2026 Feb 17;13(24):e20739. doi: 10.1002/advs.202520739 (PMC13116110; doi:10.1002/advs.202520739)
Supplement: Supplementary file 2 — Supporting File 2: advs74483‐sup‐0002‐TableS1‐S3.docx. [file ADVS-13-e20739-s002.docx]

| **Gene name** | **Primer name** | **Primer sequence** |
| --- | --- | --- |
| Tgm2 | Forward Primer | AGCCCACTCCTACCTCTCTG |
|  | Reverse Primer | TCTCCGGGATTTCAGGGGAT |
| Gas6 | Forward Primer | ATGAAGATCGCGGTAGCTGG |
|  | Reverse Primer | CCAACTCCTCATGCACCCAT |
| Il-1β | Forward Primer | TGCCACCTTTTGACAGTGATG |
|  | Reverse Primer | AAGGTCCACGGGAAAGACAC |
| Il-6 | Forward Primer | CCCCAATTTCCAATGCTCTCC |
|  | Reverse Primer | CGCACTAGGTTTGCCGAGTA |
| Tnf-α | Forward Primer | ATGGCCTCCCTCTCATCAGT |
|  | Reverse Primer | TTTGCTACGACGTGGGCTAC |
| Cd86 | Forward Primer | CTTACGGAAGCACCCACGAT |
|  | Reverse Primer | TGTAAATGGGCACGGCAGAT |
| β-Actin | Forward Primer | TGTACCCAGGCATTGCTGAC |
|  | Reverse Primer | AACGCAGCTCAGTAACAGTCC |
| Cd206 | Forward Primer | GGGCTACAGGAGAACCCAAC |
|  | Reverse Primer | AAGCTTGCCGTGCGTCTT |
| Il-10 | Forward Primer | GCCCTTTGCTATGGTGTCCT |
|  | Reverse Primer | TAGGGGAACCCTCTGAGCTG |
| Arg1 | Forward Primer | GGAACTCAACGGGAGGGTAAC |
|  | Reverse Primer | TTAGTTCTGTCTGCTTTGCTGTG |
| Il-13 | Forward Primer | CTGCAGCAAGACCGTGAGTC |
|  | Reverse Primer | GCTACAGTGAGGTAGCAGAGTTAT |
| Ccl17 | Forward Primer | AAGCTCATCTGTGCAGACCC |
|  | Reverse Primer | GGGAACAGGGACTTCTGCTC |

**Supplementary Table 1. Primer Sequences Used for qPCR in This Study**

**Supplementary Table 2: siRNA and pcDNA3.1-STAT6 Sequences Used in This Study**

| **siRNA** | **Strand** | **Sequences** |
| --- | --- | --- |
| si-Tgm2 | Sense Strand | CGGGAAUAUGUCCUUACGCAATT |
|  | Antisense Strand | UUGCGUAAGGACAUAUUCCCGTT |
| si-Gas6 | Sense Strand | CCUGGCACUGAUGGAAAUCAATT |
|  | Antisense Strand | UUGAUUUCCAUCAGUGCCAGGTT |
| pcDNA3.1-STAT6 | Forward Primer | GCTGGCTAGCGTTTAAACTTAAGCTTGCCACCATGTCTCT  GTGGGGCCTAATTTCCAAGATGTCC |
|  | Reverse Primer | CCGTCATGGTCTTTGTAGTCGGATCCCCAGCTGGGGTTGG  TCCTTAGGTCCAGGTGGGACAGTGAGATC |

**Supplementary Table 3: Primer Sequences Used for ChIP-qPCR in This Study.**

| **Gene name** | **Primer name** | **Primer sequence** |
| --- | --- | --- |
| Gas6 | Forward Primer | CACACACCUUGCAGACCAATT |
|  | Reverse Primer | CCUUCAGAAAUGGCGGACCTT |
